# Regulation of the U3-, U8-, and U13snoRNA Expression by the DEAD Box Proteins Ddx5/Ddx17 with Consequences for Cell Proliferation and Survival

**DOI:** 10.3390/ncrna2040011

**Published:** 2016-09-30

**Authors:** Hala Ismael, Simone Altmeyer, Hans Stahl

**Affiliations:** Department of Medical Biochemistry and Molecular Biology, University of Saarland, Medical Center, Building 45, 66421 Homburg, Germany; simone@misial.de (S.A.); bchsta@uniklinikum-saarland.de (H.S.)

**Keywords:** Ddx5, Ddx17, U3snoRNA, U8snoRNA, U13snoRNA, promoter, A-kinase anchor protein 9

## Abstract

Small nucleolar RNAs (snoRNAs) in cooperation with their associated proteins (snoRNPs) contribute to the maturation of ribosomal RNA, transfer RNA, and other transcripts. Most snoRNPs mediate chemical base modifications of their RNA substrates, and a few others, like those formed by the C/D snoRNAs U3, U8, and U13, are needed for the structural organization and maturation of primary transcripts. The U3-, U8-, and U13snoRNAs are encoded by autonomous genes, and our knowledge about their expression regulation is limited. In this study, a significant increase in the concentrations of U3-, U8-, and U13snoRNA after a knockdown of DEAD box proteins Ddx5/Ddx17 in HeLa cells is observed. These alterations are shown to be caused by transcriptional suppression mediated by Ddx5/Ddx17 via histone deacetylase 1 in a promoter-dependent way. The biological function of this expression control may be related to the role of Ddx5/Ddx17 in cell proliferation. The U3snoRNA is shown here to be essential for the proliferation and viability of human cells. Moreover, it was found that U3snoRNA interacts with Argonaute 2 in the RNA-induced silencing complexes (RISC), pointing to a microRNA-like function. For this reason, the 3′ untranslated region of the A-kinase anchor protein 9 (AKAP9)-mRNA could be identified as a potential target.

## 1. Introduction

The small nucleolar RNAs (snoRNAs) are non-coding RNAs with a length of 60–600 nucleotides [1], and are highly conserved within different species [2]. According to their conserved nucleotide sequence motifs, they are split into the H (ANANNA, N = any nucleotide)/ACA box snoRNAs and the C (UGAUGA/U)/D (CUGA) box snoRNAs [3]. snoRNAs are primarily found in the nucleolus associated with specific proteins to form the small nucleolar ribonucleoprotein complexes (snoRNPs) [4]. Most of them function as “guide” RNAs in the post-transcriptional modification of primary transcripts (e.g., pre-ribosomal RNAs and pre-spliceosomal RNAs) [4]. Some others, like the C/D snoRNAs U3, U8, and U13, ensure correct rRNA folding and hence cleavage by base pairing with their substrates [5,6]. The U3- and U13snoRNAs are involved in the maturation processes of the 18S rRNA [7,8], and U8snoRNA is involved in the maturation process of the 28S and 5.8S rRNA [6]. In addition, certain snoRNAs are essential for growth in the yeast *Saccharomyces cerevisiae* [1]. Some others are involved in the mediation of metabolic stress [9], the regulation of alternative splicing [10], or the negative regulation of gene expression [11,12]. In contrast to most snoRNA genes found within introns of host genes [13,14], those of U3-, U8-, and U13snoRNAs are self-developed and have their own promoters for independent transcription [15]. The knowledge about their expression regulation, however, is sparse. 

The DEAD box proteins Ddx5 (p68) and Ddx17 (isoforms p72 and p82) modulate RNA secondary structures and are involved in RNA metabolic processes like ribosome biosynthesis and splicing [16,17,18,19]. They also act as transcriptional cofactors, repressing some promoters and activating others by interaction, e.g., with the histone deacetylase 1 (HDAC1) [20] or estrogen receptor alpha, respectively [21]. Investigating the role of Ddx5/Ddx17 in the processing of 28S rRNA, we observed that these proteins also influence the cellular level of U8snoRNA in HeLa cells [19]. Here, the scope of these investigations was expanded to the expression of further snoRNAs, notably those encoded by autonomous genes. We found a distinct inhibition of U3-, U8-, and U13snoRNA transcription by Ddx5/Ddx17. The inhibition is most probably mediated by the interactions with promoter bound HDAC1. In addition, HeLa cell proliferation and viability are shown to be dependent on the presence of U3snoRNA, and thus Ddx5/Ddx17 may negatively regulate these processes by their expression control. Furthermore, we show for the first time that U3snoRNA has a microRNA (miRNA)-like function, and the A-kinase anchor protein 9 (AKAP 9)-mRNA was identified as one of its potential targets.

## 2. Results

### 2.1. Ddx5 Regulates the Expression of U3-, U8-, and U13snoRNA

**Figure 1:**
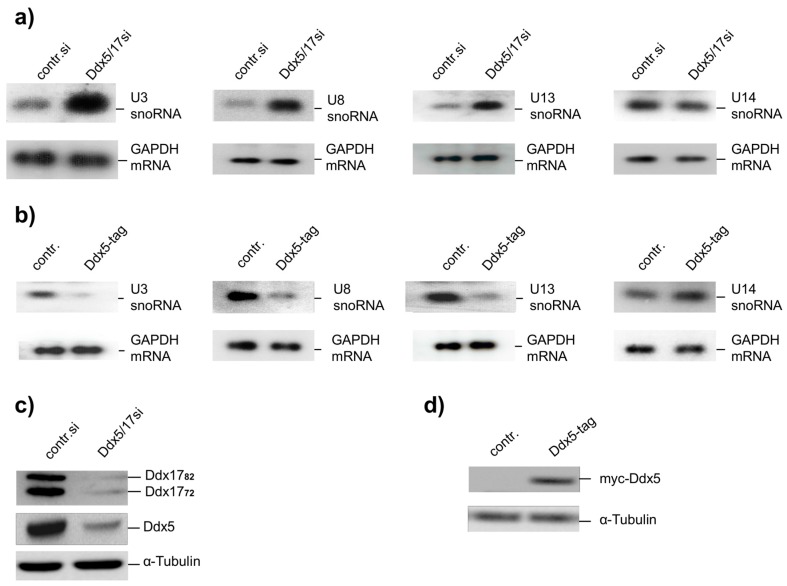
Effect of DEAD box proteins Ddx5/Ddx17 on the expression of U3-, U8-, U13-, and U14 small nucleolar RNA (snoRNA). (**a**) Northern blot analysis after knockdown of Ddx5/Ddx17. Total RNA (5 μg each) from HeLa cells transfected with indicated small interfering RNA (siRNA) for 48 h were analyzed using digoxigenin-labeled snoRNA probes and a glyceraldehyde 3-phosphate dehydrogenase (GAPDH) mRNA probe, the latter serving as an endogenous loading control; (**b**) Northern blot analysis after overexpression of Ddx5. HeLa cells were transfected with a Ddx5 expression plasmid (Ddx5-tag) for 48 h, thereafter the total RNA was extracted and analyzed as in Figure 1a; (**c**) Demonstration of efficient Ddx5/Ddx17 knockdown by Western blot analysis with α-tubulin as an endogenous loading control; (**d**) Demonstration of ectopic Ddx5 expression (Ddx5-myc) by Western blot analysis with myc antibodies. α-Tubulin was used as an endogenous loading control.

We have shown previously that the cellular concentration of U8snoRNA increases significantly with reduced levels of Ddx5 and Ddx17 in HeLa cells [19]. Here we have investigated whether this observation can be extended to the expression of other snoRNAs encoded by autonomous genes such as U3- and U13snoRNA, that are involved in the processing of the 18S pre-rRNA [22]. Because Ddx5 and Ddx17 are highly related and are both active as transcriptional regulators [20], we used a small interfering RNA (siRNA) targeting both DEAD box proteins in knockdown experiments (Figure 1c). We observed an up-regulation of the cellular content of the U3-, U8-, and U13snoRNA upon Ddx5/17 siRNA transfection increasing with time and reaching a plateau after 40–48 h (Figure 1a). In contrast, the expression of the U14snoRNA, which is also involved in the processing of pre-rRNA but encoded in the intron of the ribosomal protein S13 (Rps13) and hence not transcribed independently [1], was not affected. Off-target effects of the Ddx5/17si can be excluded by use of different siRNAs in the analysis of U8snoRNA expression in previous studies [19]. Overexpression of Ddx5 from an exogenous plasmid strongly repressed U3-, U8-, and U13snoRNA expression, whereas that of U14snoRNA was not affected (Figure 1b,d).

### 2.2. Promoter-Dependent Regulation of the U3-, U8-, and U13snoRNA Expression by Ddx5/Ddx17

**Figure 2:**
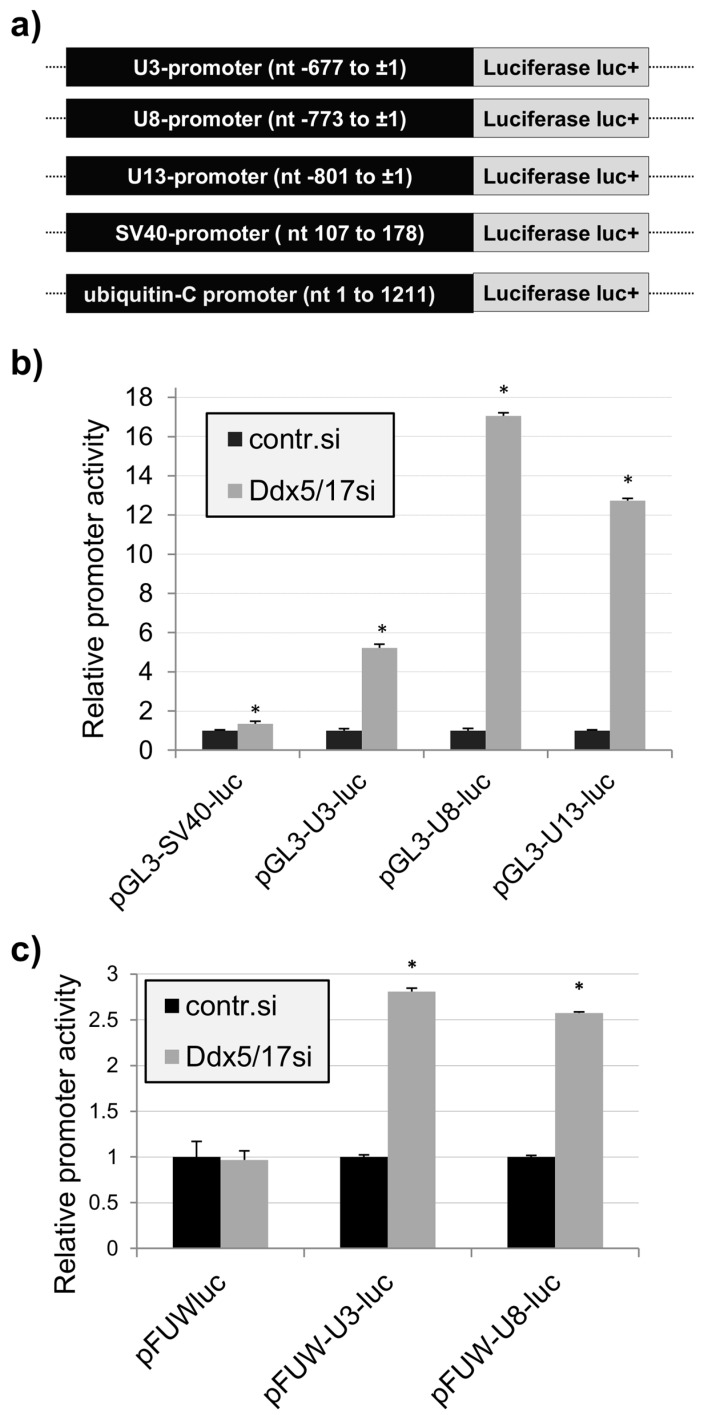
Promoter-dependent regulation of the U3-, U8-, and U13snoRNA expression by Ddx5/Ddx17. (**a**) Schematic drawing of the promoter regions (in black) used for the expression of the promoter-less firefly luciferase gene (in gray) from the respective pGL3-luc expression plasmids (pGL3-U3-luc, pGL3-U8-luc, pGL3-U13-luc, pGL3-SV40-luc) or recombinant lentiviruses (pFUWluc, pFUW-U3-luc, pFUW-U8-luc). The numbers refer to the nucleotides upstream of the respective genomic transcription start sites (±1); (**b**) and (**c**) Expression of firefly luciferase after knockdown of Ddx5/17 (Ddx5/17si) from the pGL3-luc expression plasmids in HEK293 cells (**b**) or from recombinant lentiviruses in HeLa cells (**c**) (mean ± standard deviation (SD); *n* = 3; * *p* value < 0.05). Control siRNA (contr.si) was used in parallel experiments. Measured luciferase fluorescence was converted into relative promoter activity units. The construct with the SV40 early promoter (pGL3-SV40-luc) was used as a negative control in (**b**) and that with the human ubiquitin promoter (pFUWluc) in (**c**).

Genomic regions which are expected to represent the promoters of the U3-, U8-, and U13snoRNA genes were isolated and cloned upstream of the promoter-less *Photinus pyralis* luciferase complementary DNA (cDNA) in the plasmid pGL3-basic (pGL3-luc; Figure 2a). Promoter activity of the cloned fragments and their possible dependence on Ddx5/Ddx17 were analyzed by respective luminescence measurements. The reporter assay (Figure 2b) revealed that the cloned DNA fragments, like the SV40 early region, express distinct promoter activity in HeLa cells comparable in extend to that of the SV40 early region (Figure S2). In contrast to the SV40 early region, the basic promoter activities of U3-, U8-, and U13snoRNA were elevated after knockdown of Ddx5/Ddx17 expression (up to 15-fold). Similar results were also obtained when the reporter genes were expressed after insertion into the cellular genome using a lentiviral expression system. Here the human ubiquitin C promoter was used as a control, and as expected, it did not respond to the reduction of cellular Ddx5/Ddx17 concentrations (Figure 2c). 

### 2.3. The Role of HDAC1 in the Regulation of U3-, U8-, and U13snoRNA Transcription

**Figure 3:**
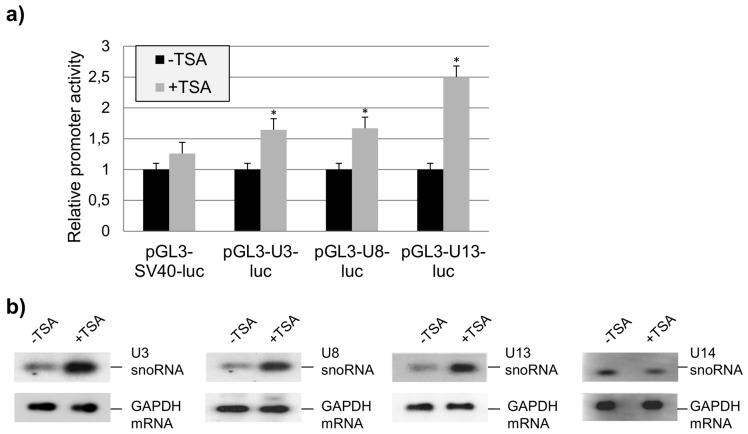
Effect of trichostatin A (TSA) on the expression of U3-, U8-, and U13snoRNA in HeLa cells. (**a**) TSA effect on the activity of isolated U3- and U8-gene promoters. Expression of firefly luciferase from plasmids pGL3-SV40-luc, pGL3-U3-luc, and pGL3-U8-luc was analyzed in cells treated with TSA (50 nM dissolved in dimethyl sulfoxide (DMSO) +TSA) or DMSO only (−TSA) in the transient luciferase reporter assay, and is shown as relative promoter activity (mean ± SD; *n* = 3; * *p* value < 0.05); (**b**) TSA effect on the expression of U3-, U8-, U13-, and U14snoRNA from endogenous genes. Total RNA (5 μg each) isolated from cells treated with TSA (50 nM in DMSO; +TSA) or DMSO only (−TSA) were analyzed by Northern blotting with GAPDH mRNA monitoring as a loading control.

**Figure 4:**
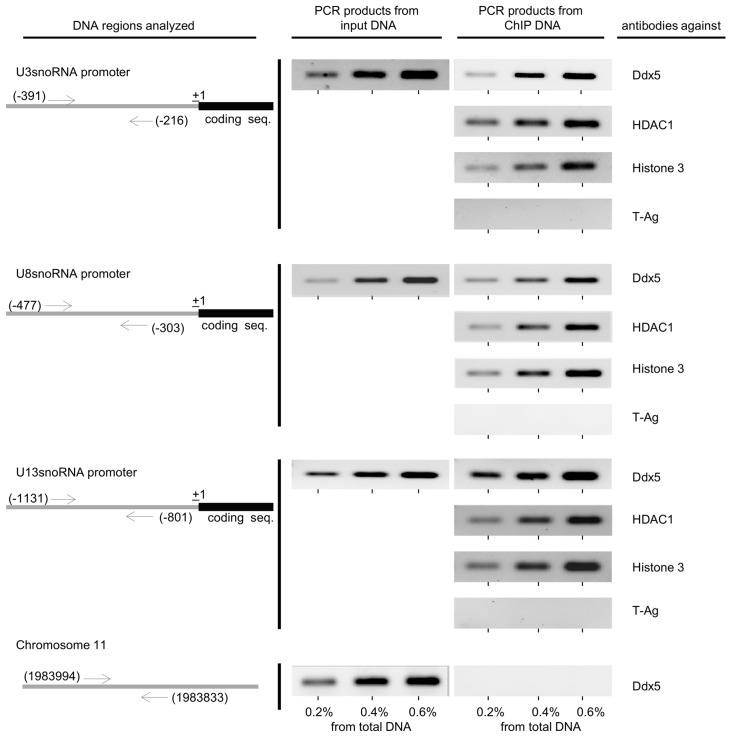
Binding of Ddx5 and histone deacetylase 1 (HDAC1) to the promoters of the U3-, U8-, and U13snoRNA genes in HeLa cells. The DNA regions analyzed by chromatin immunoprecipitation (ChIP) are schematically shown on the left, each bound by a pair of primers (arrows) showing the genomic location of their first nucleotide. A non-binding region of chromosome 11 (CHR11: 1,983,833 1,983,994) was used as a control. The antibodies used in the ChIP-precipitation analysis are shown on the right. As a control, a SV40 T-antigen-specific antibody (PAb101) was used (T-Ag). The immunoprecipitated DNA was analyzed semi-quantitatively in comparison to indicated amounts of input DNA.

Because Ddx5 and Ddx17 were previously shown to interact with HDAC1 [20], we further analyzed a possible influence of trichostatin A (TSA), an inhibitor of HDAC class I and II enzymes, on the U3-, U8-, and U13snoRNA promoter activity using the transient luciferase reporter assay. We found that the transcription of U3-, U8-, and U13snoRNA was TSA dependent (Figure 3a). Additional experiments showed that this was also true for the activity of the endogenous U3-, U8-, and U13snoRNA gene promoters but not for that of the U14snoRNA used as a negative control (Figure 3b). These results suggest that Ddx5 and a HDAC enzyme interact with the U3-, U8-, and U13snoRNA promoter, which in fact was identified by a chromatin immunoprecipitation (ChIP) assay as HDAC1 (Figure 4).

In the ChIP assay, the binding of histone 3 (H3) to the U3-, U8-, and U13snoRNA promoter regions served as a positive control. Unspecific chromatin-protein interactions were apparently not detected under the assay conditions as shown by the use of an antibody directed against the large tumor antigen of simian virus 40 (T-antigen, not present in HeLa cells). Furthermore, the missing binding of the HeLa cell chromatin region 11:1983833–1983994 (reported to be free of Ddx5 [20]) to Ddx5 further confirmed the specificity of the assay.

### 2.4. Influence of U3snoRNA Expression Level on Cell Proliferation 

**Figure 5:**
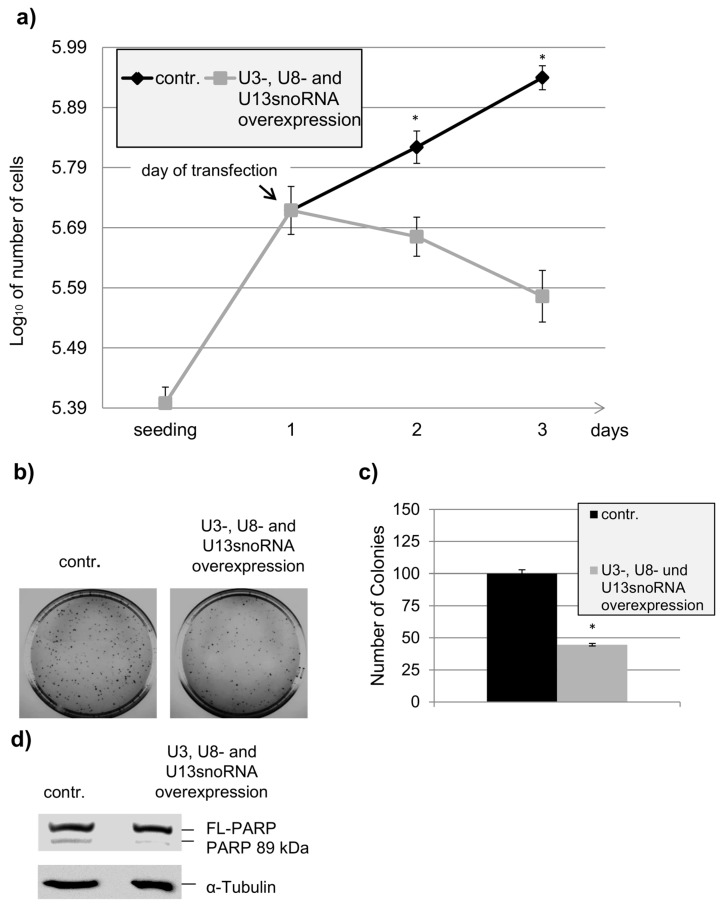
Inhibition of HeLa cell proliferation by external co-expression of U3-, U8-, and U13snoRNA. (**a**) Cell proliferation on the dependence of U3-, U8-, and U13snoRNA overexpression. Cells were transfected with a mixture of the U3-, U8-, and U13snoRNA expression plasmids (pcDNAU3, pcDNAU8, pcDNAU13), or the empty vector (pcDNA3.1(−)), and growth curves were established in a half logarithmic scale (mean ± SD; *n* = 3; * *p* value < 0.05); (**b**) An overview of the outcome of a colony forming assay performed with control cells and HeLa cells overproducing U3-, U8-, and U13snoRNA; (**c**) Quantitative analysis of the colony forming assay (mean ± SD; *n* = 3; * *p* value < 0.05). (**d**) Western blot analysis of poly(ADP-ribose) polymerase (FL-PARP) and its cleavage product (PARP 89kDa, known as an indicator of apoptotic cell death). α-Tubulin served as a loading control.

**Figure 6:**
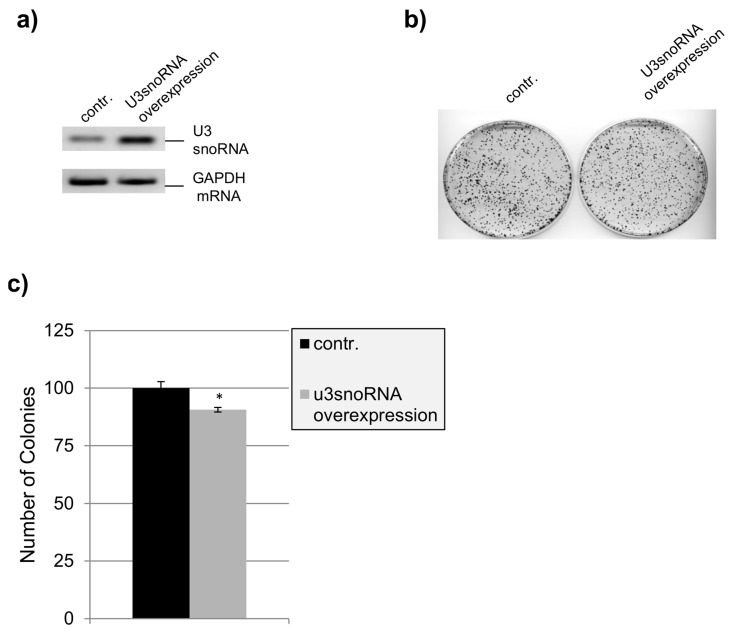
HeLa cell proliferation after external expression of U3snoRNA. Cells were transfected with the U3snoRNA expression plasmid (pcDNAU3) or the empty vector (pcDNA3.1(−)), and were harvested 48 h thereafter. (**a**) Demonstration of U3snoRNA overexpression by reverse transcription polymerase chain reaction (RT-PCR). GAPDH mRNA served as a loading control; (**b**) An overview of the outcome of a colony forming assay performed with control cells and HeLa cells overproducing U3snoRNA; (**c**) Quantitative analysis of the colony forming assay (mean ± SD; *n* = 3; * *p* value < 0.05).

Our laboratory has shown before that co-silencing of Ddx5 and Ddx17 causes inhibition of cell proliferation, perturbation of nucleolar structure, and cell death [19]. Our findings that the expression of the U3-, U8-, and U13snoRNA genes is controlled by Ddx5/Ddx17 point to some regulatory functions of these snoRNAs in cell life as well, as reported for another member of this non-coding RNA family [23]. The biological relevance of such a regulatory role of U3-, U8-, and U13snoRNA may become more obvious by altering their cellular levels. In fact, when the three snoRNAs were overexpressed simultaneously, HeLa cell proliferation was completely inhibited (Figure 5a) and the ability of the cells to form colonies was reduced by more than 50% (Figure 5b,c). Apoptosis was not observed here, as indicated by the absence of poly(ADP-ribose) polymerase (PARP) fragmentation (Figure 5d). In contrast, when the snoRNAs were overexpressed separately, proliferation of the cells seemed to not be affected, as shown here for U3snoRNA by testing the ability of the cells to form colonies (Figure 6b,c). These results suggest that the essential function of U3snoRNA for cell viability can only be titrated out by contemporary overexpression of all three snoRNAs, that seem to function in a similar manner (and with similar interaction partners), explaining at the same time their concerted expression control by Ddx5/Ddx17. 

On the other hand, U3snoRNA/RNP was shown to be an essential factor for ribosome maturation. Moreover, the knockout of U3snoRNA/RNP is known to be lethal in mouse [22]. Indeed, this is also confirmed in HeLa cells by the knockdown of U3snoRNA expression (Figure S1), which causes a strong reduction of the ability of the cells to form colonies. However, the inhibition of cell proliferation is mediated by a pro-apoptotic effect as illustrated by PARP cleavage. Interestingly, the U14snoRNA knockdown, used as a control, did not cause cell apoptosis. 

### 2.5. miRNA Function of U3snoRNA

**Figure 7:**
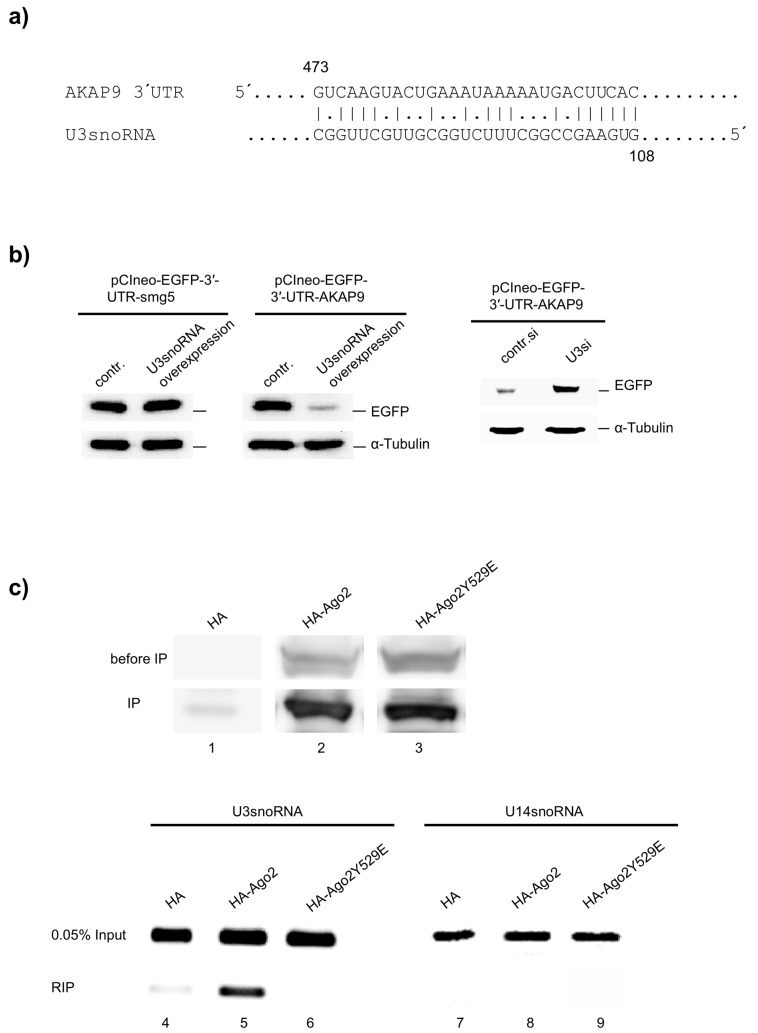
Expression regulation of a reporter construct containing the 3′ untranslated region (UTR) of the A-kinase anchor protein 9 (AKAP9) mRNA by U3snoRNA and binding of U3snoRNA to Argonaute 2 (Ago2); (**a**) Predicted complementary sequences of U3snoRNA and AKAP9 3′UTR; (**b**) Control of the expression of enhanced green fluorescent protein (EGFP) by U3snoRNA via the 3′UTR of AKAP9 mRNA linked to the EGFP-encoding cDNA (pCIneo-EGFP-3′-UTR-AKAP9). The 3′UTR of smg5 mRNA linked to the EGFP-encoding cDNA (pCIneo-EGFP-3′-smg5) was used as a control. We show the Western blot analysis of EGFP expressed from the respective constructs on dependence of U3snoRNA overexpression (left) or knockdown of U3snoRNA (right). α-Tubulin served as a loading control. Overexpression and knockdown of U3snoRNA is shown in figure 6a and Figure S1a; (**c**) Binding of U3snoRNA to Ago2. Human influenza hemagglutinin tagged Ago2 (HA-Ago2), its siRNA-binding deficient mutant (HA Ago2_Y529E), or only the HA-tag (HA) were expressed from respective plasmids for 72 h in HEK239 cells and resultant cell extracts immunoprecipitated with anti-HA antibodies. An aliquot of the cell extracts (0.05%, before immunoprecipitation (IP)) as well as an aliquot (10%) of the IP were analyzed by Western blotting using anti-HA antibodies (lanes 1–3). The remaining immunoprecipitates were analyzed for co-immunoprecipitated U3- (lanes 4–6) and U14snoRNA (lanes 7–9) by RT-PCR. An aliquot of cell extracts was co-analyzed for the snoRNAs (0.05% input).

Of particular interest in post-transcriptional gene regulation, we analyzed the U3snoRNA sequence with the DIANAT-online tool [24] to identify targets of potential U3snoRNA-derived miRNA within human genes. To capture potential miRNA regions in U3snoRNA, its sequence was split into 202 segments, each 23 nucleotides in length and moving along the sequence with one-nucleotide shift. The in silico analysis revealed several mRNAs. These mRNAs can bind to the potentially U3snoRNA-derived miRNAs with variable affinity. The mRNA of AKAP9 is one of these mRNA which represents a potential target with a score of 49 (Table S4). Figure 7a shows the predicted complementary sequences of U3snoRNA and AKAP9 3′ untranslated region (UTR).

For direct proof of the 3′UTR of AKAP9 mRNA as an U3snoRNA target, the 3′UTR sequence of AKAP9 mRNA was cloned 3′ to the coding sequence of the enhanced green fluorescent protein (EGFP), and a possible influence of the U3snoRNA on EGFP expression was investigated in HeLa cells. The cells were transfected with pCIneo-EGFP-3′-UTR-AKAP9 for 24 h, followed by splitting of the culture and transfection with either the U3snoRNA expression plasmid (pcDNAU3) or the U3snoRNAsiRNA (U3si). The results show that the cellular EGFP concentration significantly decreased after U3snoRNA overexpression (Figure 7b, left) and increased after U3snoRNA knockdown (Figure 7b, right). The expression of pCIneo-EGFP-3′-UTR-smg5 was used here as a negative control because the 3′UTR of smg5 was not identified as a predictive target in the in silico analysis. The results clearly indicate that U3snoRNA can regulate the expression of AKAP9 probably at the post-transcriptional level.

Mammalian Argonaute 2 (Ago2) specifically binds certain RNAs resulting in the assembly of catalytically active Ago2 ribonucleoprotein complexes [25]. As an indicator of a miRNA function of U3snoRNA, a possible interaction with Ago2 was investigated by RNA immunoprecipitation (RIP). For this, human influenza hemagglutinin tagged Ago2 (HA-Ago2) or its mutant (HA-Ago2Y529E) was overexpressed in HEK293 cells and immunoprecipitated from cell extract with HA-specific antibodies (Figure 7c, lanes 1–3). Co-precipitated RNA was isolated and analyzed by reverse transcription polymerase chain reaction (RT-PCR) for the presence of U3snoRNA sequences (Figure 7c, lanes 4–6). The results showed that in contrast to U14snoRNA (Figure 7c, lane 8), U3snoRNA was actually bound to Ago2 (Figure 7c, lane 5). The specificity of this interaction was verified by use of a miRNA-binding mutant of Ago2 (Ago2Y529E) in a parallel RIP experiment (Figure 7c, lane 6). Taken together, these results suggest that U3snoRNA fulfills a miRNA-like function to regulate the expression of AKAP9 and possibly additional genes.

## 3. Discussion

snoRNAs represent a class of small non-coding RNA molecules. In association with specific proteins (snoRNPs), most of them perform chemical modifications of ribosomal, transfer, and small nuclear RNAs [1]; a few, like C/D snoRNPs U3, U8, and U13 indispensably guide rRNA processing by guaranteeing correct RNA folding [3,5]. Here, we have shown that Ddx5/Ddx17 control the expression of U3-, U8-, and U13snoRNA (Figure 1a). Their expression regulation can be accomplished at the transcriptional level without regard for host genes, because these snoRNAs are encoded by autonomous genetic units (and not by intron sequences of “foreign” genes). 

We show that Ddx5/Ddx17 suppress the transcription of the U3-, U8-, and U13snoRNA genes under cellular conditions. Their knockdown, on the other hand, results in a significant increase of U3-, U8-, and U13snoRNA in HeLa cells and enhanced their promoter activities in a transient reporter assay. Both DEAD box proteins were concurrently addressed in most of the experiments performed here due to their related activities and/or functions [19]. The extent of the involvement of isolated Ddx17 in U3-, U8-, and U13snoRNA gene expression control has to be investigated. Ddx5 and Ddx17, on the other hand, have no influence on the activity of the SV40-early promoter, indicating that their regulatory functions are promoter specific. The control of the snoRNA promoter activity is also observed after integration of the reporter gene constructs into chromosomal DNA using lentiviral vehicles (Figure 2c). This suggests that it is not abolished by higher-order chromatin structures as reported for other transcription control processes [26]. 

Using a ChIP assay, we demonstrate that the suppression of the U3-, U8-, and U13snoRNA gene transcription is based on an interaction of Ddx5 with the local transcription machinery (Figure 4). Wilson et al. [20] showed that Ddx5 and Ddx17 interact with HDAC1, inhibiting transcription from the herpes virus thymidine kinase (TK) promoter in a promoter-specific manner. We have added to this the U3-, U8-, and U13snoRNA genes. The HDAC1 is shown here to be involved in the regulation of these snoRNA genes, though an involvement of additional HDACs cannot be excluded because TSA blocks mammalian HDAC class I and II [27]. HDAC1 can perform histone deacetylation only in tandem with other factors, like Ddx5, which according to our results, seems responsible for snoRNA promoter recruitment and activation of HDAC activity [28,29]. A promoter non-active HeLa cell chromatin region (chromosome 11: 1983833-1983994 [20]) served as a negative control in these assays. Binding of Ddx17 to the promoter regions of the snoRNA genes could not be traced because of the lack of suitable antibodies.

With respect to the biological relevance, we observed that the expression of U3 from an exogenous gene did not affect cell proliferation (Figure 6). However, overexpression of C/D snoRNAs was shown by others to prevent their accumulation in Cajal bodies, and thus processing of pre-rRNA impaired cell viability [30]. Interestingly, U3- and U14snoRNA are structurally and functionally highly conserved in all eukaryotes, though their genes have evolved differently in the course of evolution. In the yeast *Saccharomyces cerevisiae*, both U3- and U14snoRNA are encoded by autonomous genes, while in humans this is the case only for U3snoRNA. Here, the U14snoRNA gene is encoded by intron sequences of the gene for the ribosomal protein Rps13 [31]. Therefore, the expression of the U14snoRNA is coupled to the transcription of ribosomal protein Rps13 and not affected by Ddx5/Ddx17 (Figure 1a). In the yeast, both U3- and U14snoRNA are essential for cell proliferation [1]. In mammalian cells we have shown that this is the case only for U3snoRNA, which is also required for cell survival (Figure S1); an insufficiency of U14snoRNA seems to not impair cell viability.

Recently, it has been reported that some snoRNAs can be processed into smaller RNAs and bind to Ago2 in RNA-induced silencing complexes (RISC) [11]. In fact, some structural resemblance of miRNA precursors and snoRNAs exists [32,33], prompting us to investigate whether the snoRNAs examined here may also have miRNA functions. In search of a possible target for a miRNA-like function of U3snoRNA, we used the DIANA Lab program (DIANA-microT-CDS) which showed the highest target score for the A-kinase anchor protein 9 (AKAP9) mRNA. This prompted us to analyze the influence of U3snoRNA on the expression of AKAP9. Because of the size of the AKAP9 polypeptide, we were unable to quantify AKAP9 by Western blot analysis for this purpose, and instead used a reporter gene construct containing the 3′UTR of the AKAP9 mRNA following the coding sequence of EGFP. The results imply that the expression of AKAP9 is controlled by a miRNA derived from U3snoRNA in vivo. Overexpression of U3snoRNA, in contrast, showed no effect on the expression of the reporter construct containing the 3′UTR of smg5 mRNA used here as a negative control. To find a further indication of a miRNA-like U3snoRNA function, we searched for an interaction of U3snoRNA with proteins of the RNAi machinery, like Ago2 (Figure 7c). It is known that Ago2 specifically binds not only to mature miRNA (≈22 nt long) processed by Dicer from pre-miRNA [34], but also to miRNA precursors and longer unstructured RNAs [35,36,37]. Here, we detected full length U3snoRNA molecules in complex with Ago2, which may be processed to mature miRNA without contribution of Dicer, as has been described before for pre-miRNA produced by Drosha [35,36,37]. The specificity of the Ago2 interaction with U3snoRNA was confirmed by the parallel use of an Ago2 mutant lacking miRNA binding ability [38,39]. Furthermore, U14snoRNA did not bind to Ago2 under the same conditions in a parallel experiment (Figure 7c). This, indeed, proposes that U3snoRNA has a miRNA function, though knowledge of the regime of its processing to miRNA is missing, and a full length U3-associated function in this context cannot be excluded. preU3snoRNA is transcribed with a long 3′ tail and fitted with a 5′ monomethyl m7G cap in the nucleoplasm. In higher cells, trimethylation of the 5′ monomethyl m7G cap as well as quality control of the 3′ tail takes place thereafter in Cajal bodies. At this point, the mature snoRNA then binds to the box C/D core proteins fibrillarin [40,41], Nop56, Nop58, and 15.5K to form the early U3snoRNP [42], which is transported to the nucleolus to associate with U3-55K resulting in the mature U3snoRNP. Interestingly, the precursor U3snoRNA with monomethylated 5′ cap is unable to associate with core box C/D snoRNP proteins (fibrillarin and hNop58) and was detected not only in Cajal bodies, but also in the nucleoplasm, where Ago2 and miRNA-associated RISC are also present [43]. 

The biological implication of AKAP9 regulation by U3snoRNA is still unclear, as well as the biological function of AKAP9 [44]. However, our present study points to miRNA functions of the U3 and possibly other C/D box snoRNAs, and gives a first impression of the great potential of these snoRNAs to regulate cellular pathways.

## 4. Materials and Methods 

### 4.1. Plasmids

To generate the expression plasmid pCMVDdx5-myc, the coding sequence of pCMVDdx5wt [19] was amplified by use of the primers Ddx5-fwd and Ddx5-rev and cloned into the SfiI/XhoI restriction sites of pCMV-myc (Clontech, Heidelberg, Germany). For plasmids pGEM-U8 and pGEM-U14, see [19]. pGEM-U3, pGEM-U13, and pGEM-GAPDH were constructed by insertion of the U3, U13, or GAPDH cDNA in 3′ to 5′ orientation into the multiple cloning site of vector pGEM7zf(−) (Promega, Madison, USA) using the XhoI/BamHI, XhoI/HindIII, and XhoI/BamHI restriction sites respectively. The luciferase promoter reporter constructs pGL3-U3-luc, pGL3-U8-luc, and pGL3-U13-luc were generated by cloning of the respective DNA upstream from the snoRNA’s coding sequence in the pGL3-Basic vector of Luciferase Assay Systems from Promega using the XhoI/NcoI restriction sites. pGL3-SV40-luc was generated by inserting the SV40 early region into the same plasmid using the same restriction sites, and served as control. The expression plasmids of U3-, U8-, and U13 snoRNAs (pcDNAU3, pcDNAU8, and pcDNAU13) were generated by inserting the cDNA of these snoRNAs into the XhoI restriction site of pcDNA3.1(−) (Invitrogen, Carlsbad, CA, USA). pIRESneo-FLAG/HA-Ago2 was ordered from Addgene (Teddington, UK) (Plasmid #10822) for the expression of HA-tagged Ago2 (HA-Ago2). The Ago2 mutant plasmid pIRESneo-FLAG/HA-Ago2Y529E was generated from pIRESneo-FLAG/HA-Ago2 using the QuikChange II Site-Directed Mutagenesis Kit (Stratagene, La Jolla, CA, USA) according to the manufacturer’s instructions and used for the expression of the siRNA-binding deficient mutant (HA-Ago2Y592E). pCIneo-EGFP-3′-UTR-AKAP9 was constructed by inserting the 3′UTR of the AKAP9 mRNA into the pCIneo-EGFP 3′ to the stop signal of EGFP and upstream of the SV40 late poly(A) signal using the BspEI/NotI restriction sites [45]. For the pCIneo-EGFP-3′-UTR-smg5 plasmid, see [45]. Sequences of the primers used are given in Table S1. Correct structures of created DNA constructs were confirmed by DNA sequencing.

### 4.2. Cell Culture, Cell Transfection, and Exogenous Gene Expression

Hela cells (ATCC, CCL-2) and HEK293 cells (ATCC, CRL-1573) were obtained from the American Type Culture Collection (ATCC). HEK293-T (ATCC, CRL-11268) were a gift from Gerald Thiel, Saarland University, Medical Center, Homburg, Germany. The cells were cultured at 37 °C and 5% CO_2_ in Dulbecco’s modified Eagle’s medium (DMEM) with 10% fetal calf serum (FCS). Transfections of DNA and siRNA were performed for 48–72 h using jetPEI (Peqlab, Erlangen, Germany) and Interferin (Peqlab), respectively, according to the manufacturer’s protocol (for siRNA target sequences, see Table S2; AllStars Negative Control siRNA from Qiagen (Hilden, Germany) was used as a control). Cells were treated directly or 24 h after transfection with TSA (50 nM) dissolved in dimethyl sulfoxide (DMSO) for 30 h. The EGFP-3′-UTR-reporter assay was performed by transfection of pCIneo-EGFP-3′-UTR-AKAP9 or pCIneo-EGFP-3′-UTR-smg5 in HeLa cells for 24 h followed by splitting of the cells and transfection with the expression plasmid of U3snoRNA (pcDNAU3), as described previously [46]. 

### 4.3. Lentiviral Gene Transfer

To produce the transfer vectors pFUW-U3-luc and pFUW-U8-luc, the pGL3-U3-luc and pGL3-U8-luc were digested with XhoI and NcoI and the resultant promoter fragments were cloned into the PacI/BglII and PacI/BamHI restriction sites of the lentiviral transfer vector pFUWluc [47] upstream of the luciferase gene. Viral particles were produced by triple transfection of HEK293-T cells with the transfer vectors, the gag-pol-rev packaging plasmid, and the env plasmid encoding vesicular stomatitis virus glycoprotein (kind gifts of Gerald Thiel, Saarland University, Medical Center, Homburg, Germany), as described previously [48]. Used primer sequences are provided in Table S1.

### 4.4. Reporter Assays

For transient expression, cells were transfected with pGL3-U3-luc, pGL3-U8-luc, pGL3-U13-luc, or pGL3-SV40-luc for 24 h and were then treated with Ddx5/17siRNA for 48 h. For lentiviral reporter gene expression, cells were infected with the viruses containing the respective reporter construct for 24 h and then treated with the indicated siRNAs for 48 h. Extracts of cells were prepared using reporter lysis buffer (Promega) and analyzed for luciferase activities as described by Thiel et al. [49]. In each case, luciferase activities were correlated to total protein concentrations and normalized to control groups, whereby variations in control groups were less than 15%.

### 4.5. RNA Isolation

Total RNA was isolated from cells using the RNeasy Mini Kit (Qiagen) according to the manufacturer’s protocol. DNase treated total RNA (1 μg) was transcribed into cDNA using random hexamer primers and the Transcriptor High Fidelity cDNA Synthesis Kit (Roche, Mannheim, Germany), and was used for investigating the knockdown and the overexpression of U3snoRNA.

### 4.6. Northern Blot Analysis

Negative strand full-length U3-, U8-, U13-, U14snoRNA, and GAPDH mRNA were obtained by in vitro transcription of the corresponding plasmids (pGEM-U3, pGEM-U8, pGEM-U13, pGEM-U14, and pGEM-GAPDH) in the presence of digoxigenin. Northern blot analyses were performed as described elsewhere [17] and the signals detected according to the Roche DIG Northern Starter Kit manual. 

### 4.7. Antibodies

Mouse anti-tubulin (#05-829) was from Millipore (Darmstadt, Germany). For monoclonal mouse antibody C10 and polyclonal rabbit anti-human Ddx17 antibody, see [17,19]. Mouse monoclonal anti-c-myc (clone 9E10) and rat anti-HA (clone 3F10) antibodies were purchased from Roche. Mouse anti-GFP (MAB3580) was from Upstate (New York, NY, USA). For monoclonal antibodies PAb101, see [50]. Rabbit polyclonal anti-HDAC1 was from Santa Cruz (Heidelberg, Germany). Rabbit monoclonal anti-histone H3 (D2B12XP, ChIP formulated) as well as anti-PARP antibodies were from Cell Signaling Technology (Frankfurt, Germany); horseradish peroxidase-conjugated goat anti-mouse (A4416) and goat anti-rabbit (A0545) antibodies were from Sigma (Taufkirchen, Germany).

### 4.8. RNA Immunoprecipitation (RIP)

All steps were performed at 4 °C. HEK293 cells were transfected for 72 h with pIRESneo-FLAG/HA Ago2, pIRESneo-FLAG/HA Ago2_Y529E, or pCMV-HA (used as a negative control), and were harvested by centrifugation in phosphate-buffered saline (PBS) and resuspended in lysis buffer (20 mM 4-(2-hydroxyethyl)-1-piperazineethanesulfonic acid (HEPES)–NaOH, pH 7.5, 10 mM NaCl, 2 mM MgCl_2_, 1 mM ethylene glycol-bis(β-aminoethyl ether)-*N*,*N*,*N*′,*N*′-tetraacetic acid (EGTA), 0.35% Nonidet P-40, and 0.2% Na-deoxycholate, pH 8.6) supplemented with 1 mM phenylmethylsulfonyl fluoride (PMSF), 2 mM NaVO_3_, and Complete Protease Inhibitor ethylenediaminetetraacetic acid (EDTA)-free (Roche) in the presence of RiboLock RNase Inhibitor (Fermentas, St. Leon-Rot, Germany) and Phosphatase Inhibitor Cocktail (Sigma). Cell lysates were centrifuged at 14,000× *g* for 10 min. The supernatants, pre-cleared with protein A-sepharose beads, were incubated with protein A-sepharose-bound anti-HA (Roche) overnight. RNA was purified from the eluent by proteinase K digestion, phenol–chloroform-RNA extraction, and ethanol precipitation. 

### 4.9. Chromatin Immunoprecipitation (ChIP) 

The cell chromatin was extracted and aliquots (10 μg) were immunoprecipitated with respective antibodies (10 μg) by using the SimpleChIP Enzymatic Chromatin IP Kit (Cell Signaling Technology), according to the manufacturer’s instructions. Recovered DNA was analyzed by semi-quantitative PCR with primers specific for the indicated promoter regions. The sequences of used primers are provided in Table S3. DNA amplification was performed with the Kappa Robust PCR Kit according to the manufacturer’s protocol (Roche) and as described previously [19]. PCR products were analyzed by 1.2% agarose gel electrophoresis and ethidium bromide staining.

### 4.10. Cell Viability and Proliferation 

Cell viability was determined 24 h and 48 h after co-transfection of respective plasmids using the trypan blue technique. For colony formation assays, 5 × 10^3^ cells were plated in 10-cm dishes the day after transfection, and 7–14 days thereon resulting colonies were fixed with 5% glutaraldehyde and stained with 1% crystal violet in PBS. Growth kinetics were determined in triplicates after transfection of respective plasmids, and cells were counted in duplicates. The resulting mean values are given.

## Supplementary Materials

The following are available online at www.mdpi.com/2311-553X/2/4/11/s1, Table S1: Primers used for construction of plasmids; Table S2: Used small interfering RNAs (siRNAs); Table S3: Primers used for chromatin immunoprecipitation (ChIP); Table S4: Putative targets of U3snoRNA segments functioning as microRNA (miRNA); Figure S1: Indication of apoptosis by U3snoRNA knockdown; Figure S2: Promoter activity of the isolated U3-, U8-, and U13snoRNA gene promoters in comparison to that of the SV40 early region. The original images for all the gels presented in this work can be found in a separate supplementary PDF file at www.mdpi.com/2311-553X/2/4/11/s2.
