# Supplementary material for: Regulation of the U3-, U8-, and U13snoRNA Expression by the DEAD Box Proteins Ddx5/Ddx17 with Consequences for Cell Proliferation and Survival
Source: Noncoding RNA. 2016 Sep 30;2(4):11. doi: 10.3390/ncrna2040011 (PMC5831926; doi:10.3390/ncrna2040011)
Supplement: Supplementary file 1 [file ncrna-02-00011-s001.pdf]

Supplementary Materials: Regulation of the U3-, U8-, and U13snoRNA Expression by the DEAD Box Proteins Ddx5/Ddx17 with Consequences for Cell Proliferation and Survival

Hala Ismael, Simone Altmeyer and Hans Stahl

Supplementary Tables

Table S1. Primers used for construction of plasmids.

Constructs	Forward Primer 5′-3′	Reverse Primer 5′-3′
pCMVDdx5-myc	CGAATTCGGTCGACCATGTCGGG TTATTCGAGTGA	CGCTCGAGCTATTGGGAATATCC TGTTGG
pGEM-U3	GGCCTCGAGAAGACTATACTTT CAGGGATCAT	GCCCTCGAGACCA CTCAGACCGC GTTCTCTCCC TCTCA
pGEM-U13	GCCCTCGAGATCCTTTTGTAGTT CATGAGCGTGAGATT	CGGAAGCTTGGTCAGACGGGTA ATGTGCCCACGT
pGEM-GAPDH	GCCCTCGAGATGGGGAAGGTGA AGGTCGGAGTC	GCGGATCCTTACTCCTTGGAGGC CATGTGGG
pGL3-U3-luc	GCCCCTCGAGCTGCAGCTTTGAA CTCAGCCAGCTTCCCTG	GGCCCATGGAACGGCCTCCTCG CACACCTCTGCTAGG
pGL3-U8-luc	GCCCCTCGAGCCACTTGCCTTGG TAGTGCCCTTCCAGCTG	GGCCCATGGACAGACAAACAGC CGACATTCTGCACTCAGTG
pGL3-U13-luc	GCCCCTCGAGCTGACTGCAGCAC CAGAAGG	GGCCCATGGCATCTTATAGTTGA CATTAAGGAGTGC
pGL3-SV40-luc	GCCCCTCGAGCAGTTAAGGGTGT GGAAAGTCCCCAG	GGCCCCATGGCTGGAATAGCTC AGAGGCCGAGGCG
pcDNAU3	GGCCTCGAGCTAATTAAGACTAT ACTTTCAG	GGCCTCGAGCCCGAGATCGCGC GCCACTG
pcDNAU8	GGCCTCGAGCTAACTATCGTCAG GTGGGATAATCCTT	CTTGGCCTCGAGAATCAGACAG GAGCAATCAGGGTG
pcDNAU13	GGCCTCGAGCTAACTATCCTTTT GTAGTTCATGAGCGTGATGATT	CTTGGCCTCGAGGGTCAGACGG GTAATGTGCCCACGT
pIRESneo- FLAG/HA-Ago2Y529E	GGCAAGACGCCCGTGGAGGCCG AGGTCAAGCG	CGCTTGACCTCGGCCTCCACGGG CGTCTTGCC
pCIneo-EGFP-3′ -UTR-AKAP9	CAACTCCGGATAAGCCAGAAAG CAGATAGAAGGAGTTTG	ACCTGCCCCCCGGCCGCGTCTG ATCATTTCTAATTCATGATTACA GCCGG
pFUW-U3-luc	GCCTTAATTAAGTCTGGGCGGAG GGAGGAAAGGG	TAGCAGATCTAACGGCCTCCTCG CACACCT
pFUW-U8-luc	GCCTTAATTAACCACTTGCCTTG GTAGTGCCCTTCCAGCTG	GGCGGATCCACAGACAAACAGC CGACATTCTGCACTCAGTG

Table S2. Used small interfering RNAs (siRNAs).

siRNA	Target Sequence 5′-3′
Ddx5/Ddx17si	AAGGCTAGATGTGGAAGATGT
U3si	CTGAACGTGTAGAGCACCGAA
U14si	ATTGGTTGCCAGACATTCGCA

Table S3. Primers used for chromatin immunoprecipitation (ChIP).

Promoter	Forward Primer 5′-3′	Reverse Primer 5′-3′
U3snoRNA promoter	GCCTGGGCTGGTGTCGAACT CATA	AAGAGGTTTAAAACCGGGGG GGGGGGCATGCT
U8snoRNA promoter	CGTCTTGAGTCTGGGATTAT CCGC	GATACCAAGTATCTTACGGTC TGCAGG
U13snoRNA promoter	GTCTTGGAGGAGGGGGCGA GT	TGTAAGACTCTTTTACTCCCT GAGAGTCC

Table S4. Putative targets of U3snoRNA segments functioning as microRNA (miRNA). To capture potential miRNA regions in U3snoRNA, its sequence was split into 202 segments, 23 nucleotides in length and moving along the sequence with one-nucleotide shift. Results of the DIANA-T Analysis with a threshold value above 19 are shown.

Sequence of U3snoRNA Segments	Target with Scores > 19.0
UUAAGACUAUACUUUCAGGGAUC	SOX6/(5) 27.55<br>DCP2 23.16
GACUAUACUUUCAGGGAUCAUUU	ZFPM2 = Fog2 interagiert mit p300
ACUAUACUUUCAGGGAUCAUUUC	ZFPM2 36,8
AUACUUUCAGGGAUCAUUUCUAU	VGLL3 colon carcinoma related protein 23,08
CUUUCAGGGAUCAUUUCUAUAGU	CREB 3L3 (CREB-H)33
UUUCAGGGAUCAUUUCUAUAGUG	CREB 3L3 (CREB-H)32
AUCAUUUCUAUAGUGUGUUACUA	Dystonin (DST) 22/AKAP 19
UCAUUUCUAUAGUGUGUUACUAG	AKAP9 22,9/(DST)
CAUUUCUAUAGUGUGUUACUAGA	AKAP9 21,5/KLF12 24.19
AUUUCUAUAGUGUGUUACUAGAG	KLF12 21.68
UAGUGUGUUACUAGAGAAGUUUC	Integrin-β-binding protein 20
AGUGUGUUACUAGAGAAGUUUCU	Integrin-β-binding protein 39
GUGUGUUACUAGAGAAGUUUCUC	Integrin-β-binding protein
UAGAGAAGUUUCUCUGAACGUGU	NTRK3 23
AGAAGUUUCUCUGAACGUGUAGA	EVC (DWF1) Ellis-van Creveld syndrome protein 33
GAAGUUUCUCUGAACGUGUAGAG	EVC (DWF1) 46
AAGUUUCUCUGAACGUGUAGAGC	EVC (DWF1) 44<br>Microtubule-actin crosslinking factor 1<br>MACF1 24
AGUUUCUCUGAACGUGUAGAGCA	EVC (DWF1) 40 + div
GUUUCUCUGAACGUGUAGAGCAC	EVC (DWF1) 32 + div + AKAP9 26/MACF1 25
UUUCUCUGAACGUGUAGAGCACC	DST AKAP9 MACF1
UUCUCUGAACGUGUAGAGCACCG	MACF1 22
UCUCUGAACGUGUAGAGCACCGA	MACF1 19
AGCACCGAAAACCACGAGGAAGA	Cadherin 23 21.6
GAAAACCACGAGGAAGAGAGGUA	GABA receptor 24 ROD
AAAACCACGAGGAAGAGAGGUAG	ROD regulator of differentiation
CGAGGAAGAGAGGUAGCGUUUUC	PolyA ribose polymerase PARP11
GAGGAAGAGAGGUAGCGUUUUCU	PARP11
AGGAAGAGAGGUAGCGUUUUCUC	PARP11
GGAAGAGAGGUAGCGUUUUCUCC	PARP11
GAAGAGAGGUAGCGUUUUCUCCU	PARP11 26
AAGAGAGGUAGCGUUUUCUCCUG	Actin binding LIM protein
AGAGAGGUAGCGUUUUCUCCUGA	
GAGAGGUAGCGUUUUCUCCUGAG	
AGAGGUAGCGUUUUCUCCUGAGC	extrem

GAGGUAGCGUUUUCUCCUGAGCG	extrem
AGGUAGCGUUUUCUCCUGAGCGU	Fibrillin
GUUUUCUCCUGAGCGUGAAGCCG	DST
UUUUCUCCUGAGCGUGAAGCCGG	AKAP9/MACF1/onecut2
UUUCUCCUGAGCGUGAAGCCGGC	Pericentrin 32 MACF1
UUCUCCUGAGCGUGAAGCCGGCU	DST
UCUCCUGAGCGUGAAGCCGGCUU	Importin 9 22 import of ribosomal proteins
UGAGCGUGAAGCCGGCUUUCUGG	SV2 β 33
GAGCGUGAAGCCGGCUUUCUGGC	Laminin SV2 receptor
GGCUUUCUGGCGUUGCUUGGCUG	MACF1 26.14 AKAP9 23 CDK6 22.5
GCUUUCUGGCGUUGCUUGGCUGC	AKAP9 24 DST 32.25 MACF1 26.6 CDK6
CUUUCUGGCGUUGCUUGGCUGCA	DST MACF1 AKAP9
CUGCAACUGCCGUCAGCCAUUGA	NTRK3
UGCAACUGCCGUCAGCCAUUGAU	NTRK3
GCAACUGCCGUCAGCCAUUGAUG	NTRK3
CAACUGCCGUCAGCCAUUGAUGA	NTRK3
CGUUCUUCUCUCCGUAUUGGGGA	AKAP9 20.5 MDN1 19.11
GUUCUUCUCUCCGUAUUGGGGAG	AKAP9 49.83
UUCUUCUCUCCGUAUUGGGGAGU	AKAP9 44.17 eIF4G3
UCUUCUCUCCGUAUUGGGGAGUG	AKAP9 eIF4G3
CUUCUCUCCGUAUUGGGGAGUGA	div. + AKAP20
UUCUCUCCGUAUUGGGGAGUGAG	div. zinc finger protein
CCGUAUUGGGGAGUGAGAGGGAG	UBE2E3 UBE2E4P ubiquitin conjugating enzymes
UUGGGGAGUGAGAGGGAGAGAAC	
UGGGGAGUGAGAGGGAGAGAACG	
GGGGAGUGAGAGGGAGAGAACGC	GNG13
GGGAGUGAGAGGGAGAGAACGCG	GNG13
AGUGAGAGGGAGAGAACGCGGUC	BOK32
GUGAGAGGGAGAGAACGCGGUCU	BOK32
UGAGAGGGAGAGAACGCGGUCUG	GRWD1 glutamate rich WD repeat containing protein A
AGAGGGAGAGAACGCGGUCUGAG	NADH
GAGGGAGAGAACGCGGUCUGAGU	NADH
AGGGAGAGAACGCGGUCUGAGUG	
GGAGAGAACGCGGUCUGAGUGGU	PAX2

Supplementary Figures

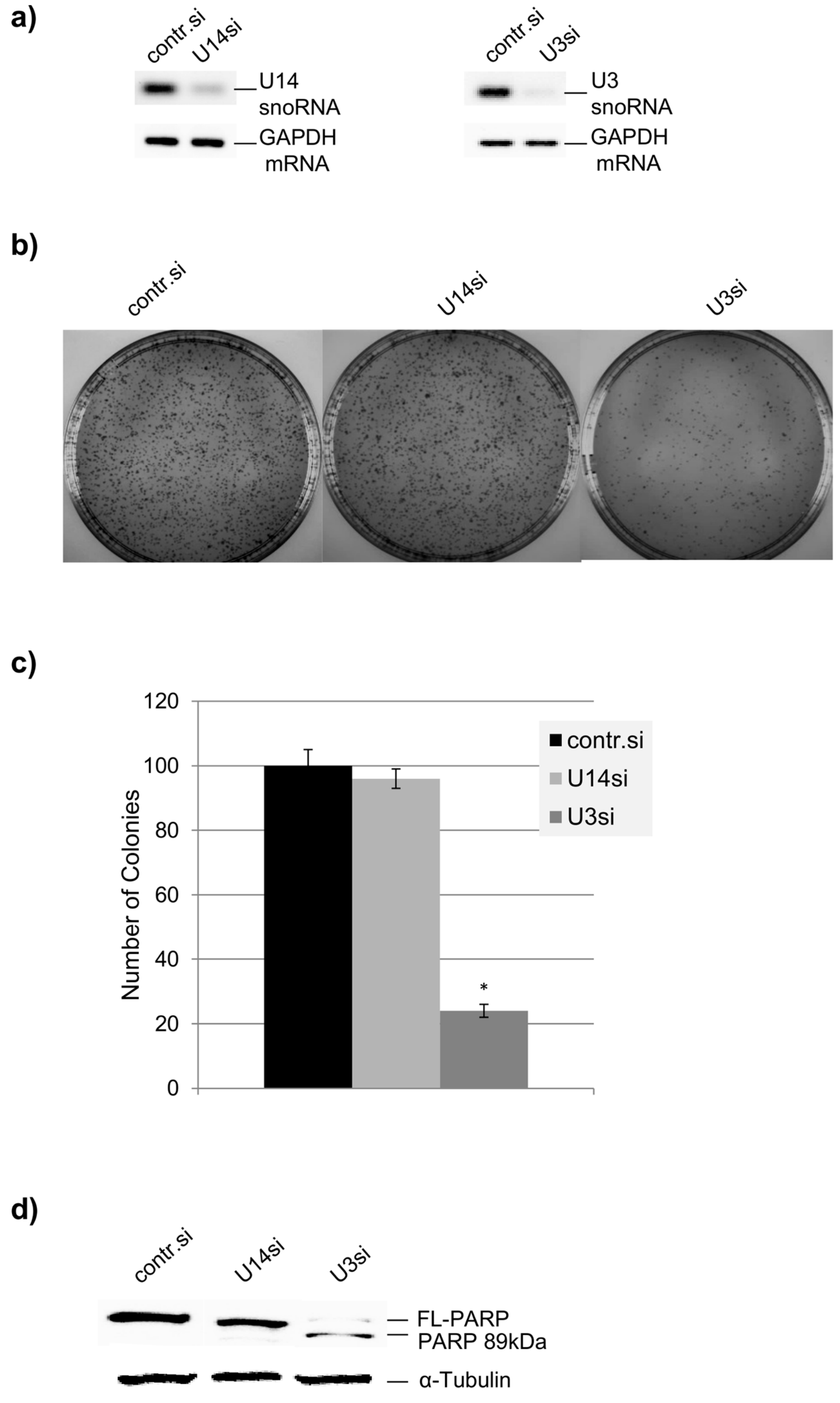

Figure S1. Indication of apoptosis by U3snoRNA knockdown. HeLa cells were transfected with control small interfering RNA (siRNA) (contr.si), U14snoRNAsiRNA (U14si), or U3snoRNAsiRNA (U3si) and harvested 72 h thereafter. (**a**) Demonstration of the U14- and U3snoRNA knockdown. 1 μg of total RNA was analyzed by reverse transcription polymerase chain reaction (RT-PCR) with glyceraldehyde 3-phosphate dehydrogenase (GAPDH) mRNA serving as a loading control; (**b**) An overview of the outcome of a colony forming assay performed with cells transfected with control siRNA (contr.si), U14snoRNAsiRNA (U14si), or U3snoRNAsiRNA (U3si); (**c**) Quantitative analysis of the colony forming assay (mean ± standard deviation (SD); $n = 3$; * p value < 0.05); (**d**) Western blot analysis of poly(ADP-ribose) polymerase (FL-PARP) cleavage into the 89 kDa fragment (PARP 89 kDa) after small nucleolar RNA (snoRNA) knockdown. α-Tubulin served as a loading control.

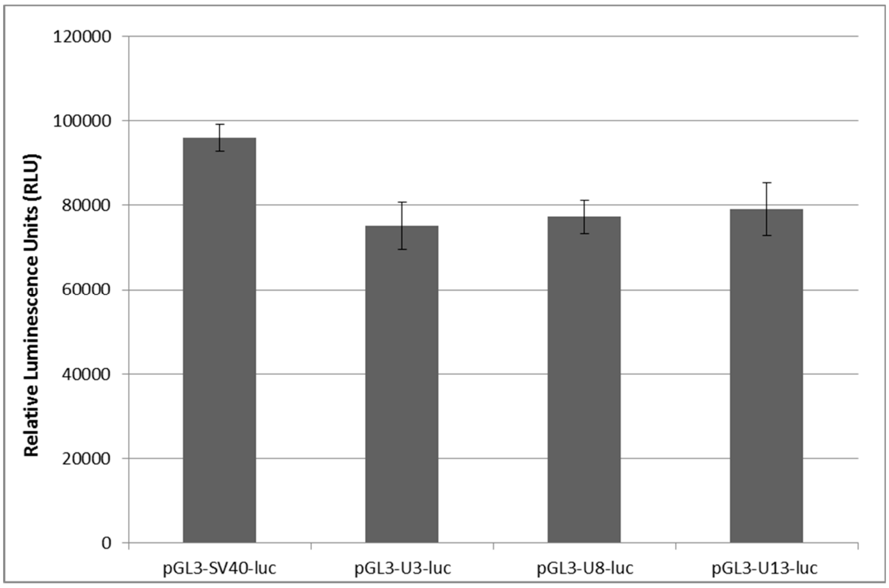

Figure S2. Promoter activity of the isolated U3-, U8-, and U13snoRNA gene promoters in comparison to that of the SV40 early region. For details of the constructs see Figure 2a. Expression of firefly luciferase is expressed as luminescence units (mean ± SD; n = 3).
