# Supplementary material for: Regulation of the U3-, U8-, and U13snoRNA Expression by the DEAD Box Proteins Ddx5/Ddx17 with Consequences for Cell Proliferation and Survival
Source: Noncoding RNA. 2016 Sep 30;2(4):11. doi: 10.3390/ncrna2040011 (PMC5831926; doi:10.3390/ncrna2040011)

Supplementary Materials: Regulation of the U3-, U8-, and U13snoRNA Expression by the DEAD Box Proteins Ddx5/Ddx17 with Consequences for Cell Proliferation and Survival

Hala Ismael, Simone Altmeyer and Hans Stahl

Raw data from Figure 1

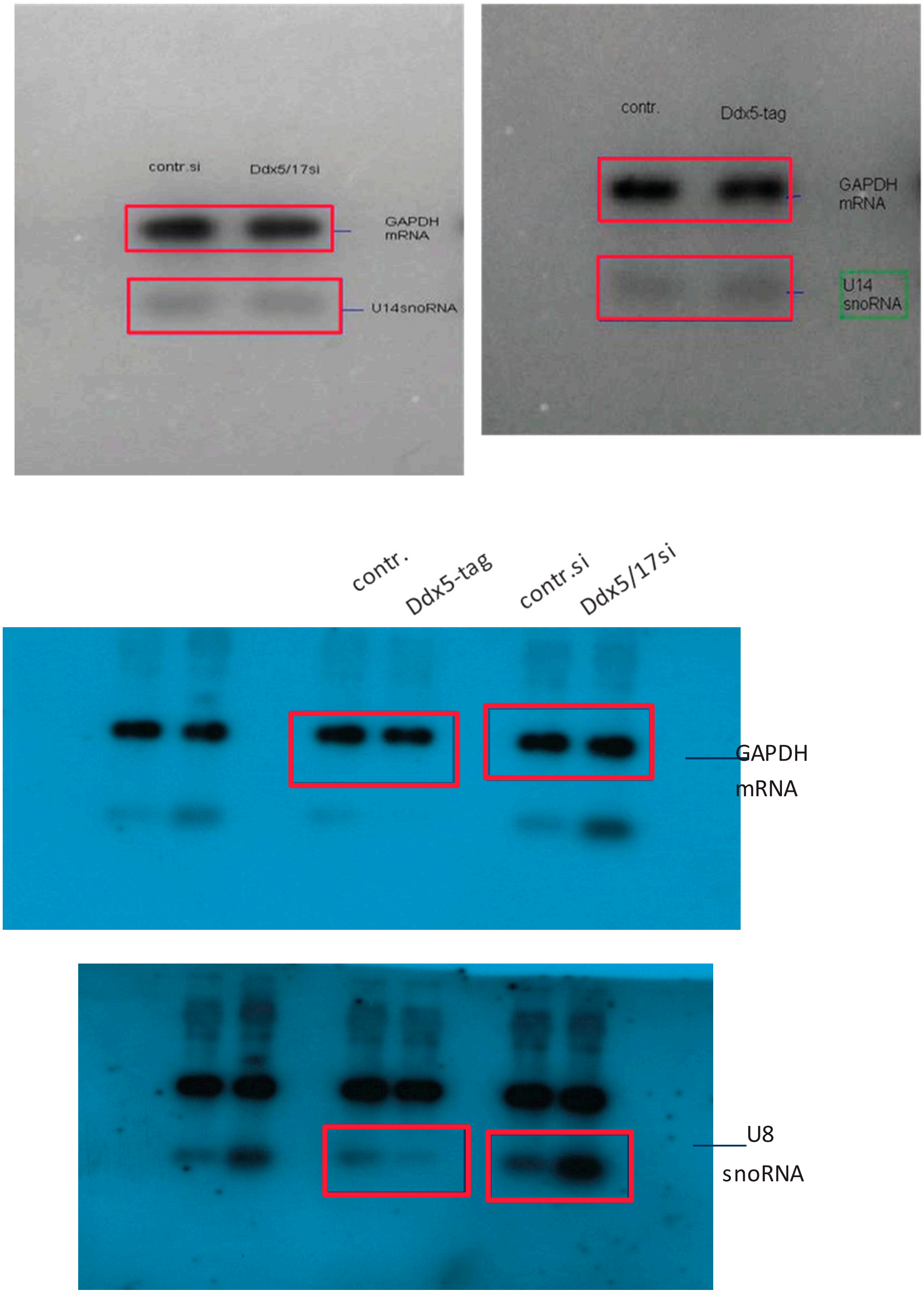

Raw data from Figure 1

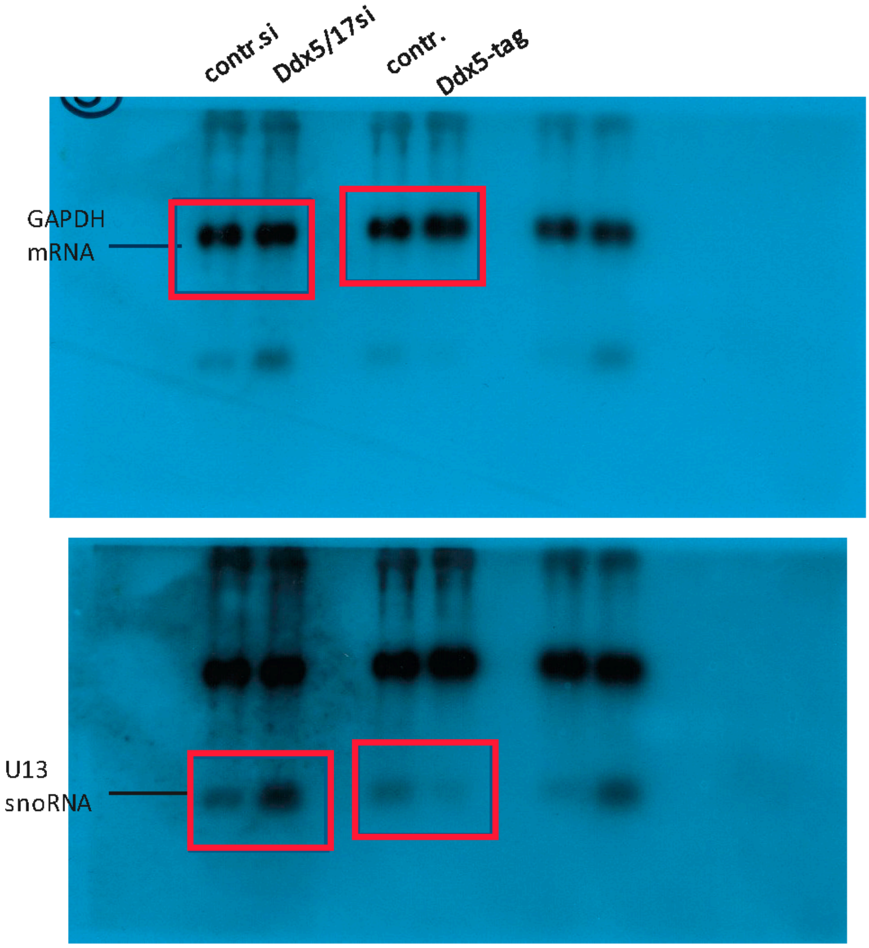

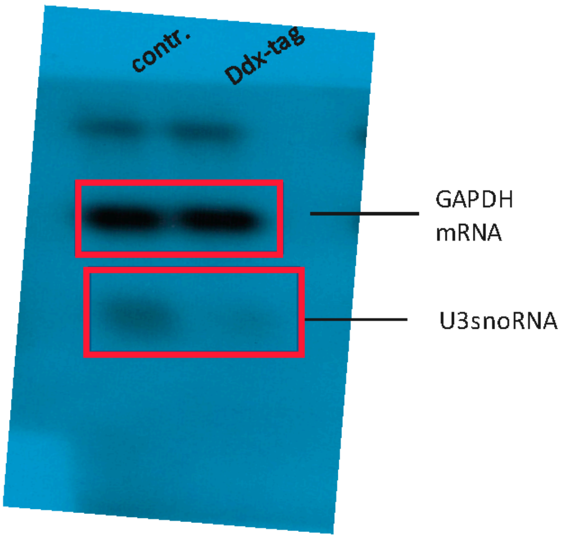

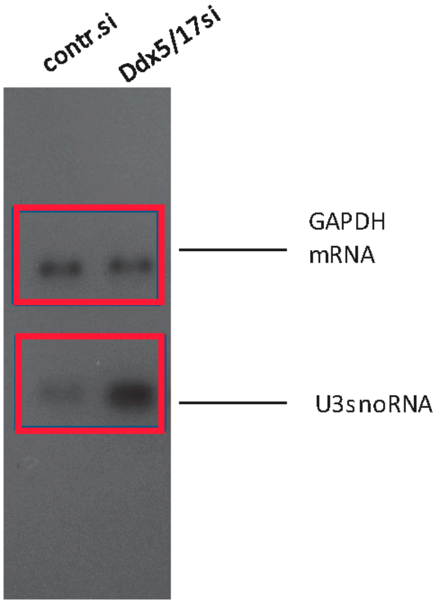

Raw data from Figure 1

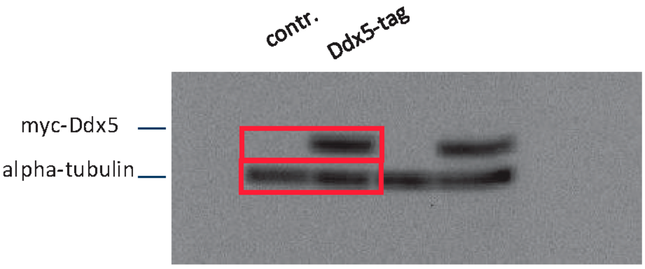

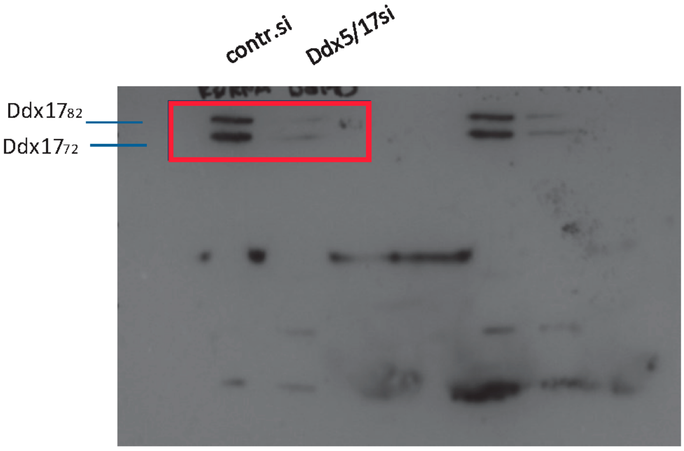

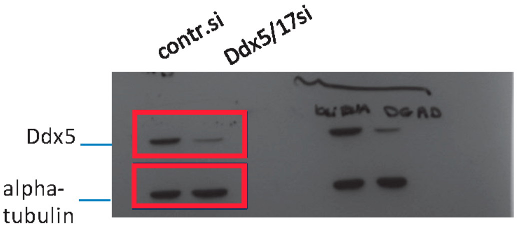

Raw data from Figure 3

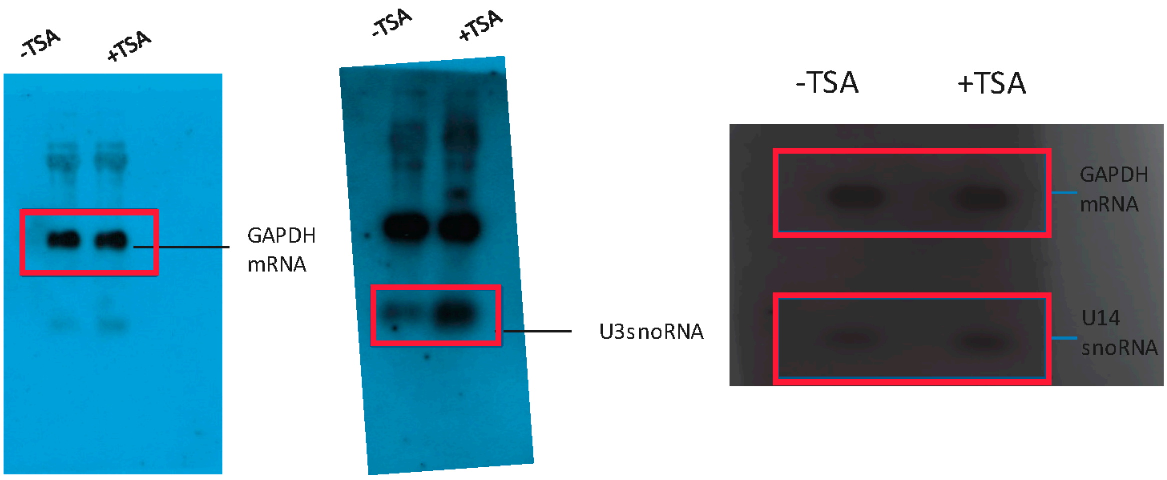

Raw data from Figure 3

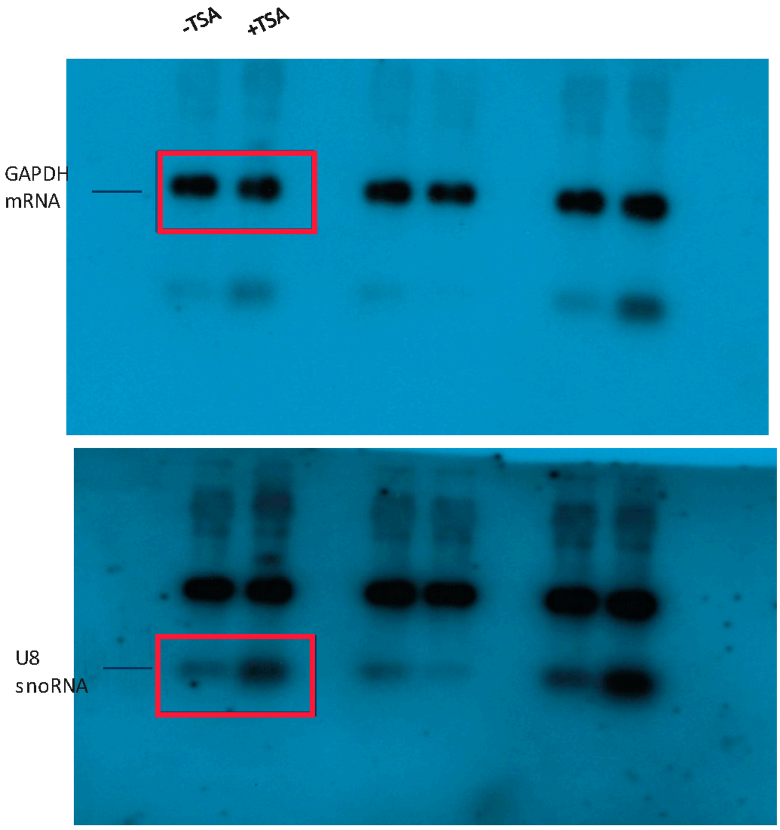

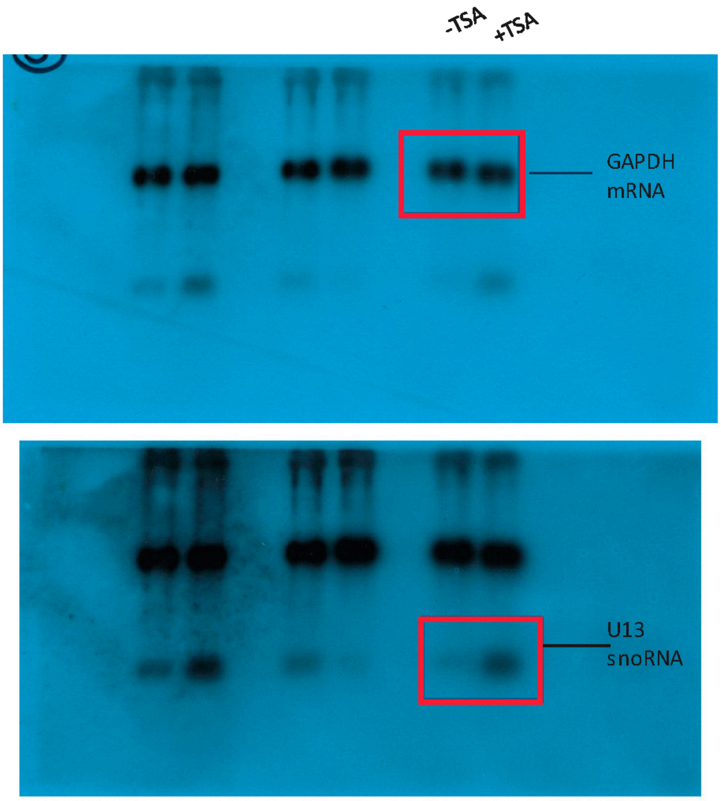

Raw data from Figure 4

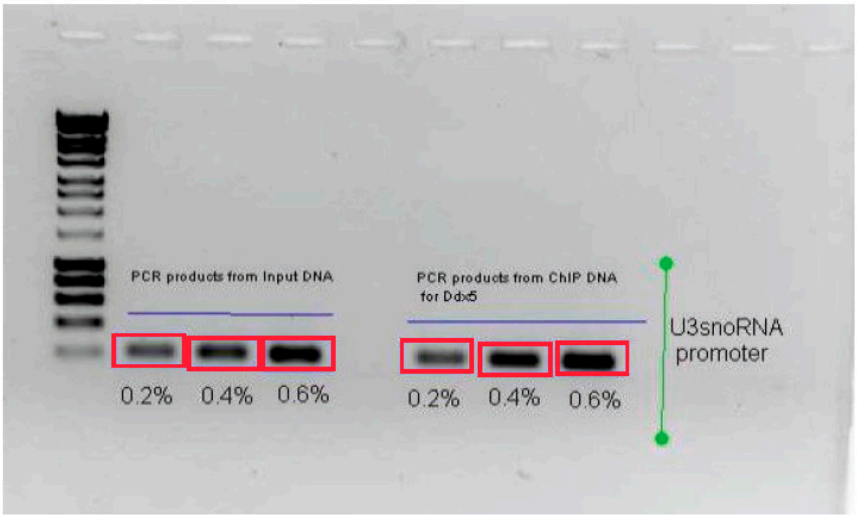

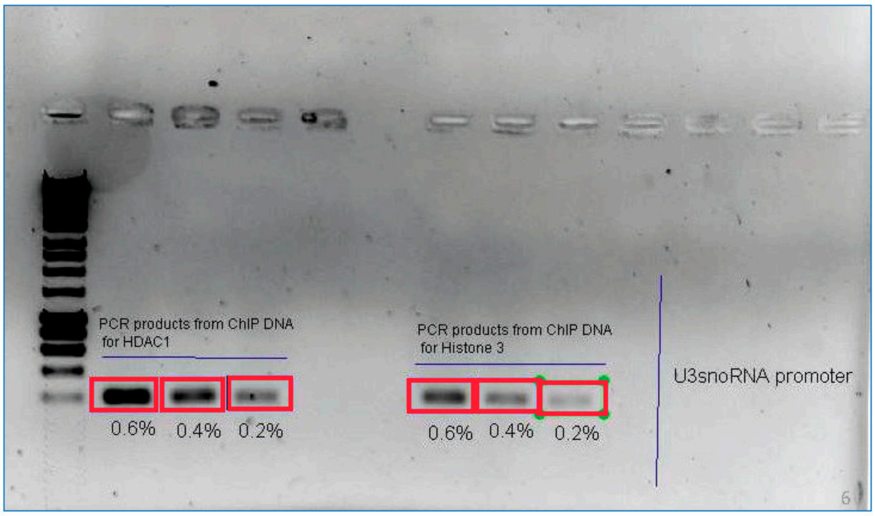

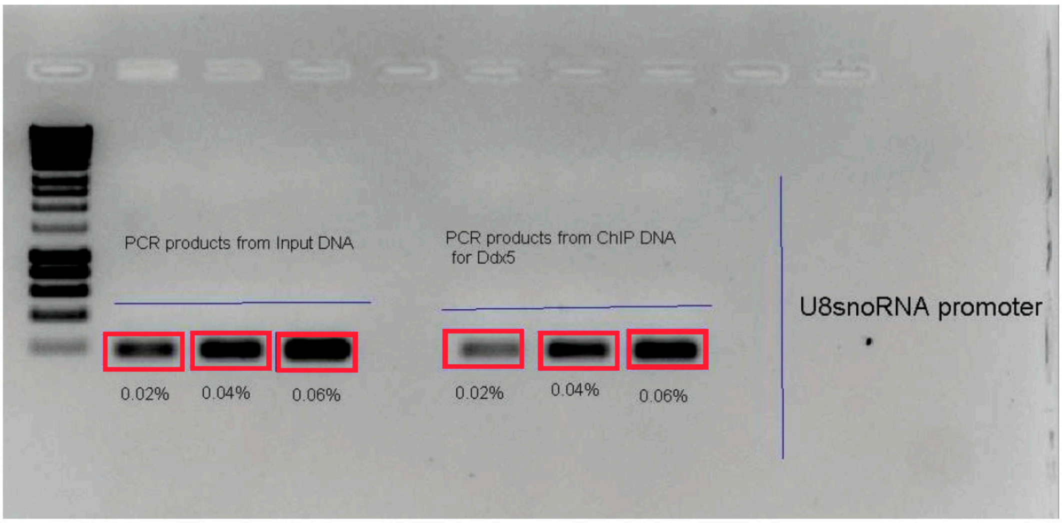

Raw data from Figure 4

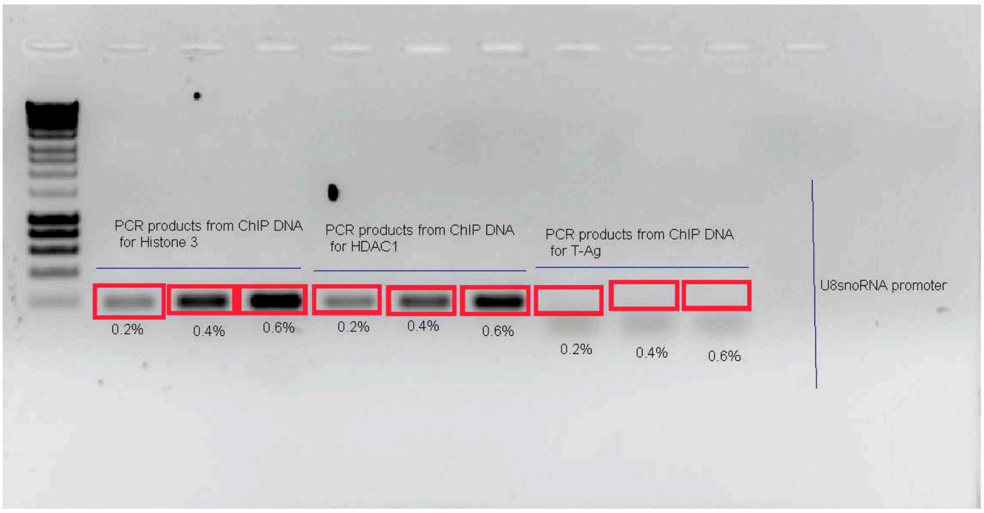

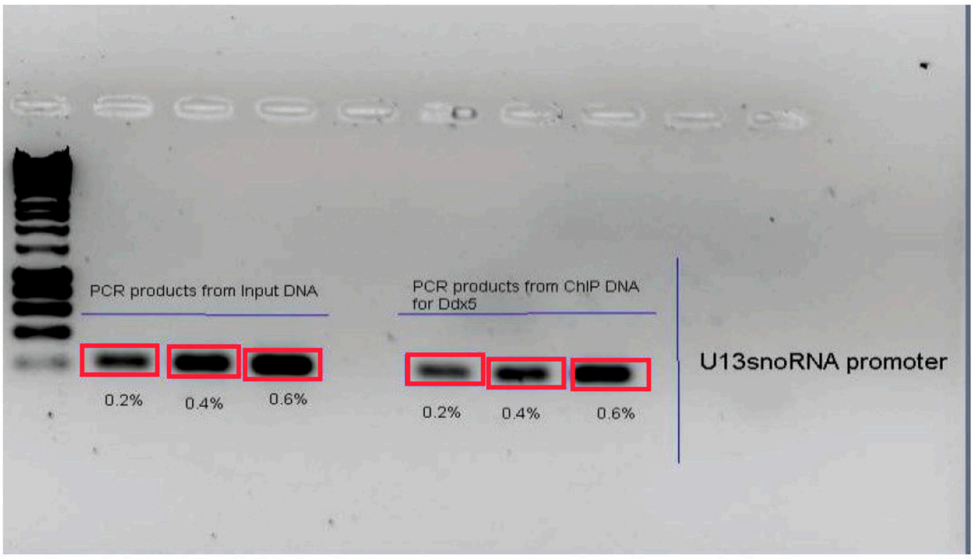

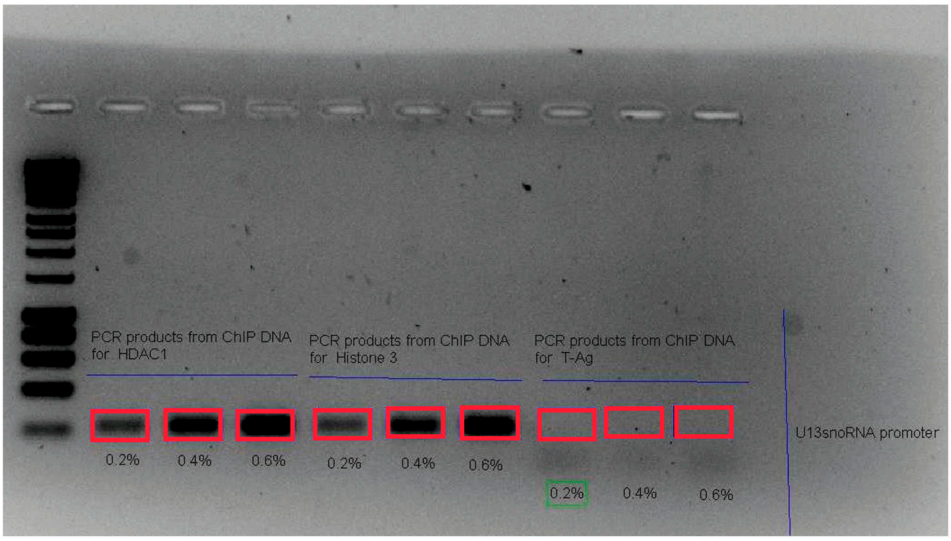

Raw data from Figure 4

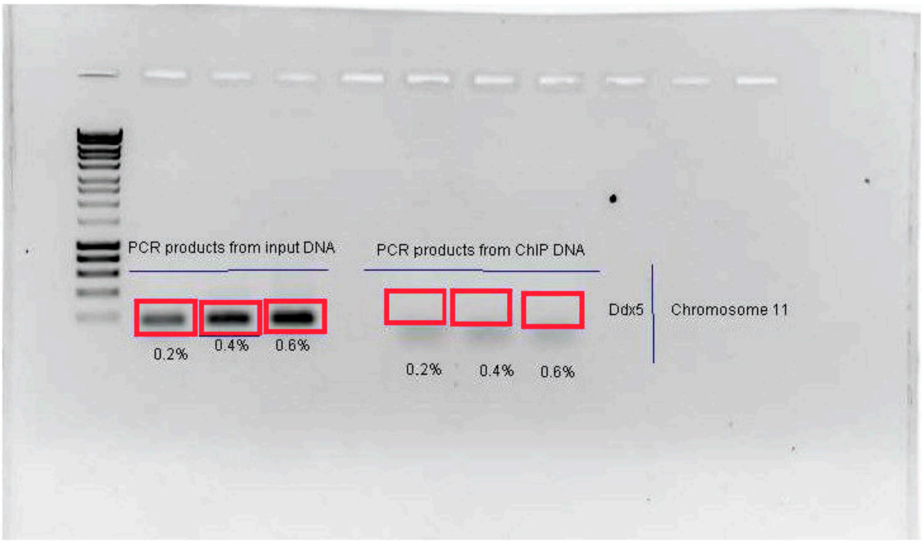

Raw data from Figure 5

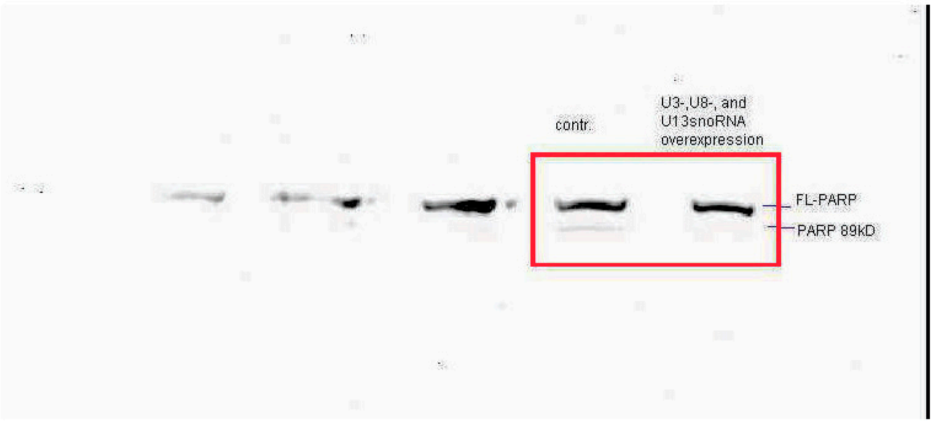

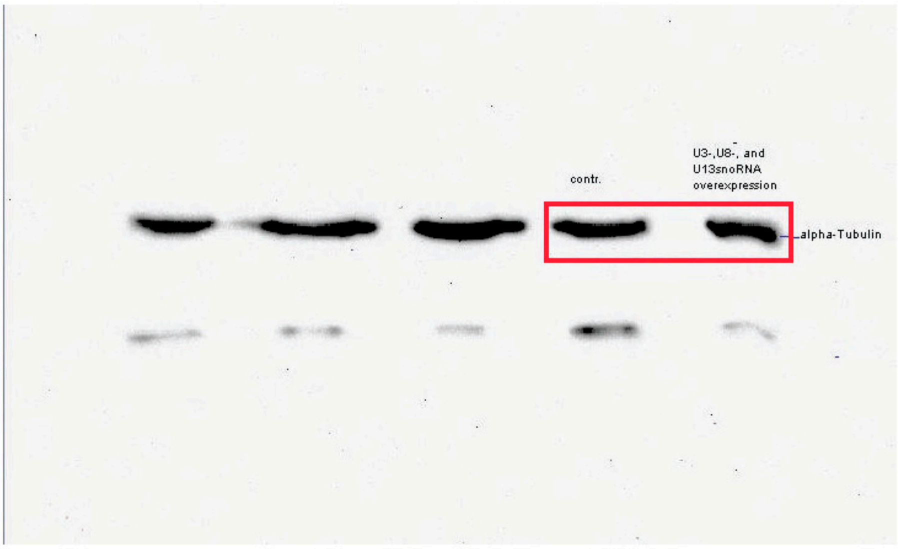

Raw data from Figure 6

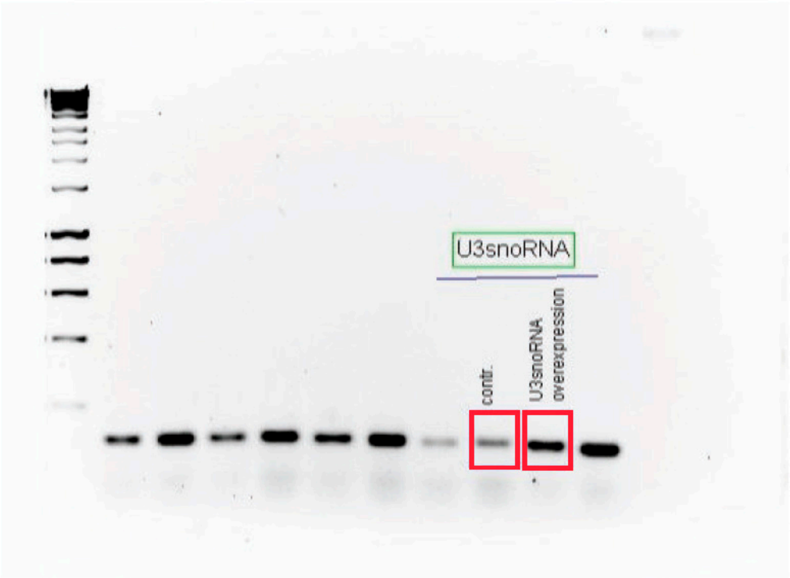

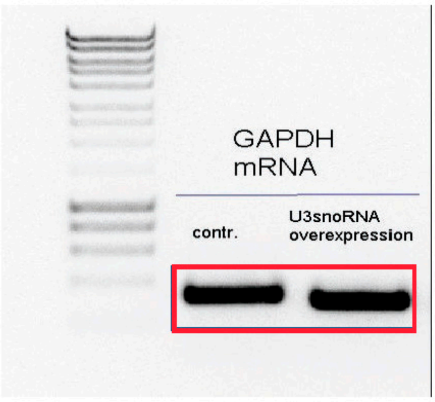

Raw data from Figure 7

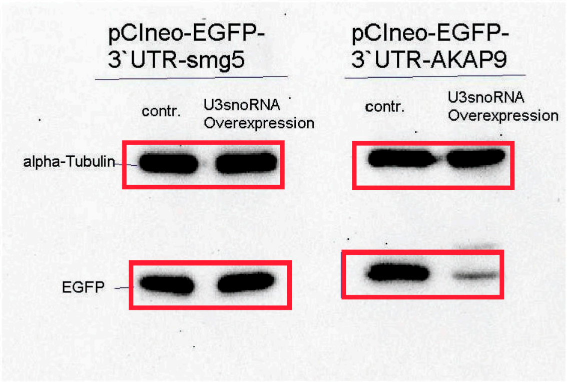

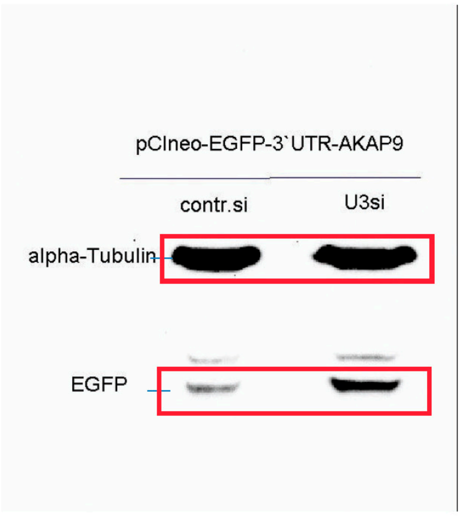

Raw data from Figure 7

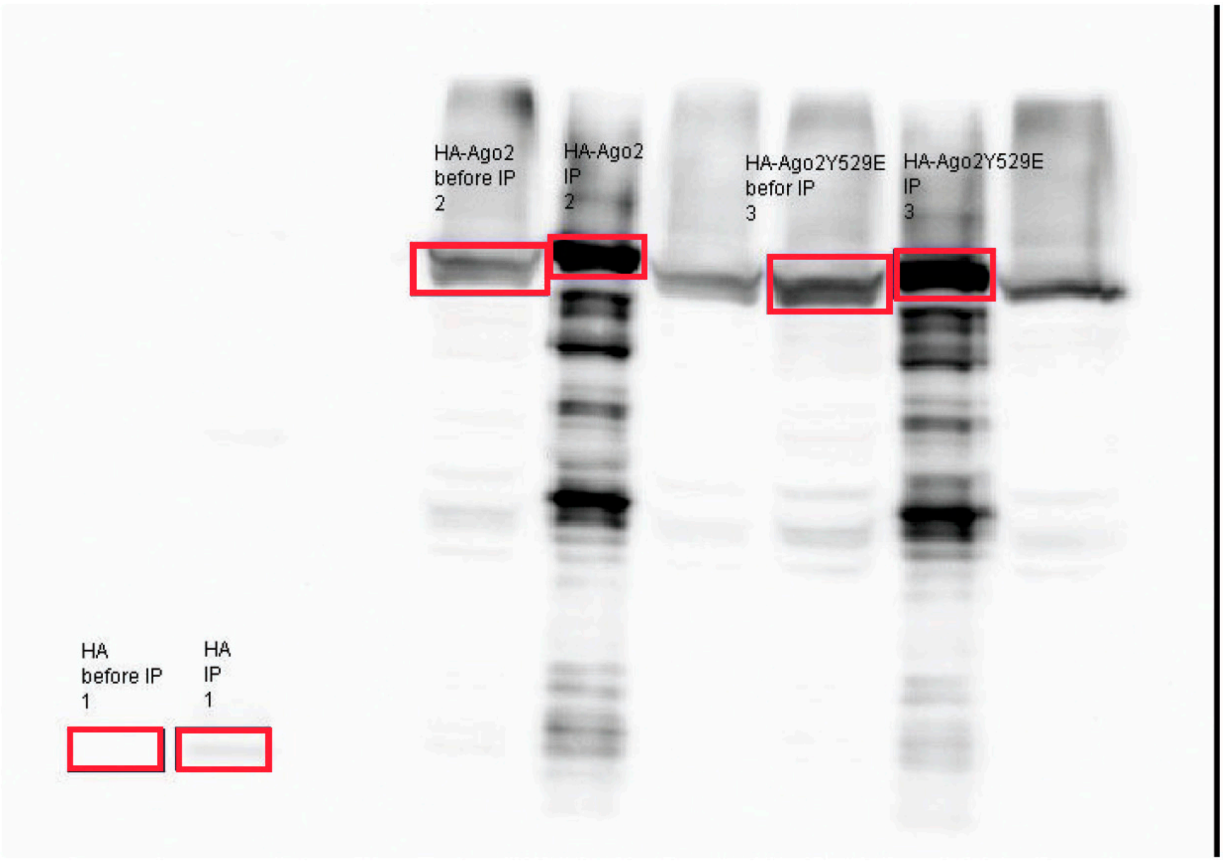

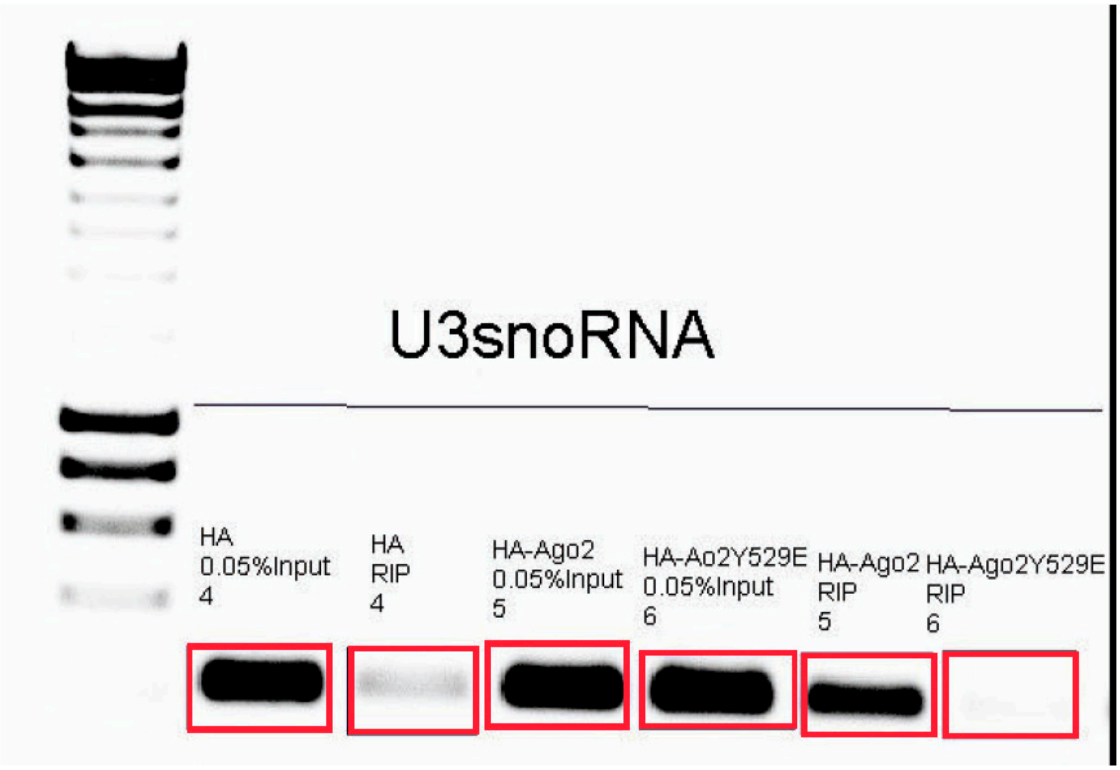

Raw data from Figure 7

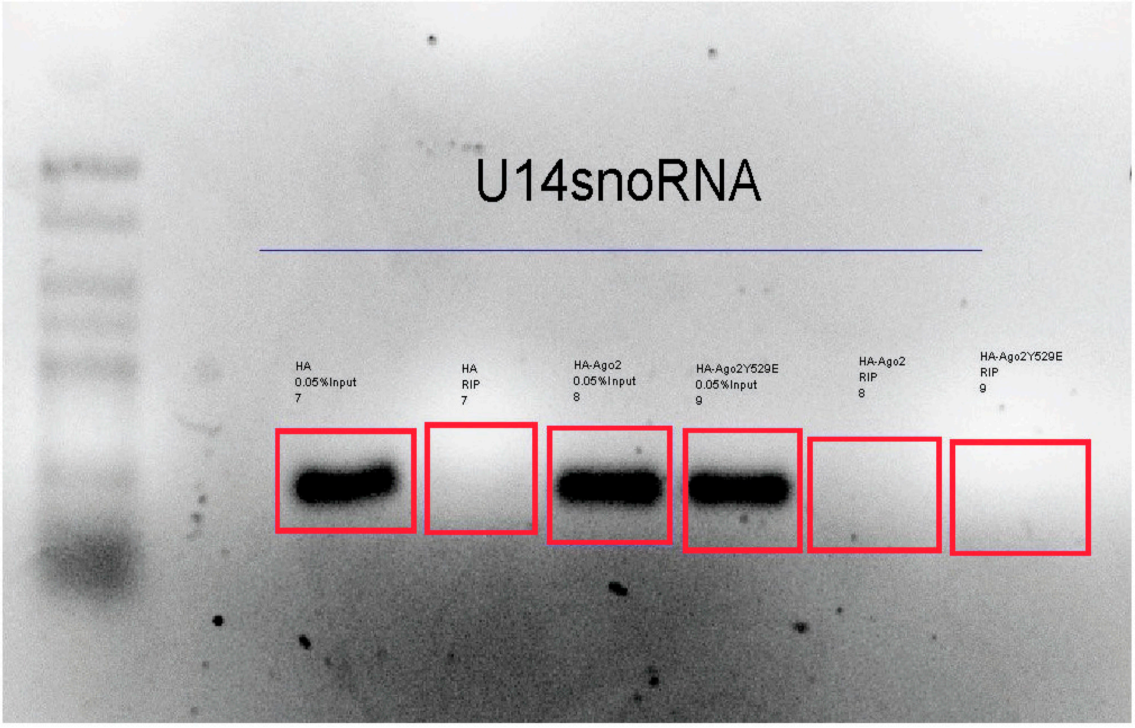

Supplement: Supplementary file 2 [file ncrna-02-00011-s002.pdf]
